# Cell-Free Approach for Non-canonical Amino Acids Incorporation Into Polypeptides

**DOI:** 10.3389/fbioe.2020.01031

**Published:** 2020-09-28

**Authors:** Zhenling Cui, Wayne A. Johnston, Kirill Alexandrov

**Affiliations:** Synthetic Biology Laboratory, School of Biology and Environmental Science, Queensland University of Technology, Brisbane, QLD, Australia

**Keywords:** genetic code expansion, genetic code reprogramming, cell-free protein synthesis, non-canonical amino acids, amber suppression, codon reassignment

## Abstract

Synthetic biology holds promise to revolutionize the life sciences and biomedicine via expansion of macromolecular diversity outside the natural chemical space. Use of non-canonical amino acids (ncAAs) via codon reassignment has found diverse applications in protein structure and interaction analysis, introduction of post-translational modifications, production of constrained peptides, antibody-drug conjugates, and novel enzymes. However, simultaneously encoding multiple ncAAs *in vivo* requires complex engineering and is sometimes restricted by the cell's poor uptake of ncAAs. In contrast the open nature of cell-free protein synthesis systems offers much greater freedom for manipulation and repurposing of the biosynthetic machinery by controlling the level and identity of translational components and reagents, and allows simultaneous incorporation of multiple ncAAs with non-canonical side chains and even backbones (N-methyl, D-, β-amino acids, α-hydroxy acids etc.). This review focuses on the two most used *Escherichia coli*-based cell-free protein synthesis systems; cell extract- and PURE-based systems. The former is a biological mixture with >500 proteins, while the latter consists of 38 individually purified biomolecules. We delineate compositions of these two systems and discuss their respective advantages and applications. Also, we dissect the translational components required for ncAA incorporation and compile lists of ncAAs that can be incorporated into polypeptides via different acylation approaches. We highlight the recent progress in using unnatural nucleobase pairs to increase the repertoire of orthogonal codons, as well as using tRNA-specific ribozymes for *in situ* acylation. We summarize advances in engineering of translational machinery such as tRNAs, aminoacyl-tRNA synthetases, elongation factors, and ribosomes to achieve efficient incorporation of structurally challenging ncAAs. We note that, many engineered components of biosynthetic machinery are developed for the use *in vivo* but are equally applicable to the *in vitro* systems. These are included in the review to provide a comprehensive overview for ncAA incorporation and offer new insights for the future development in cell-free systems. Finally, we highlight the exciting progress in the genomic engineering, resulting in *E. coli* strains free of amber and some redundant sense codons. These strains can be used for preparation of cell extracts offering multiple reassignment options.

## 1. Introduction

Proteins are the central functional constituents in all living organisms that are formed by ribosomal biosynthesis using genetically determined sequences of the 20 canonical amino acids with the rare exception of two recently discovered proteinogenic amino acids selenocysteine (Sec) (Bock et al., 1991) and pyrrolysine (Pyl) (Srinivasan et al., 2002). Genetic encoding of non-proteinogenic or non-canonical amino acids (ncAAs) in living organisms poses many challenges due to the requirement for additional transport and biosynthetic components. Using multiple rounds of negative and positive screening, the Schultz group pioneered engineering orthogonal tRNAs and aminoacyl-tRNA synthetases (aaRSs) for incorporation of tyrosine analogs into proteins *in vivo* (Wang et al., 2001). This pioneering work utilizing amber codon suppression opened up a new field of genetic code expansion (Wang et al., 2006b; Liu and Schultz, 2010). Subsequent efforts resulted in demonstration of ribosomal site specific incorporation of >200 chemical entities into proteins endowing them with novel physical, chemical, and biological properties (Neumann, 2012; Dumas et al., 2015; Xiao and Schultz, 2016).

While the focus of the initial efforts has been on utilization of the self-replicating nature of living organisms to produce polypeptides with ncAAs, recently cell-free systems have become progressively more utilized for this purpose. As a testimony to the practical value of this approach, two successful biotechnology companies, Sutro Biopharma Inc. and PeptiDream Inc., utilize ncAA incorporation into polypeptides using the *E. coli* cell-free protein synthesis systems (CFPS). At present, these companies are estimated to have approximately $220M and $4.9B market value, respectively.

In this review, we first compare the crude (S30) and fully reconstituted (PURE) *E. coli* cell-free protein synthesis systems (Table 1) and discuss their strengths and weaknesses. We then delineate the strategies and components required for the template-directed ncAA incorporation, including creation of vacant codons and synthesis of acylated tRNAs. The recent invention of unnatural nucleic acid base pairs can be used as orthogonal codons to dramatically expand the genetic code *in vivo* and *in vitro*. We summarize advances in engineering of the translational machinery (tRNAs, aaRSs, elongation factors, and ribosomes). Finally, we highlight the exciting progress in the genome engineering field that led to *E. coli* strains free of amber codons and some redundant sense codons and their use for preparation of more efficient CFPS.

**Table 1:** Comparison of the crude and PURE *E.coli* cell-free protein synthesis systems.

	**Crude CFPS**	**PURE CFPS**
Developed	Year 1964	Year 2001
Cost	< $0.05/reaction	$10/reaction
Composition	Crude extract (>500 proteins) + necessary factors	36 purified proteins+ tRNAs + ribosomes + necessary factors
Energy source	Versatile energy sources including variants that take advantage of cellular metabolism	Creatine phosphate/creatine kinase ATP regeneration system
Flexibility	Partial control of translational machinery	Full control of translational machinery
Linear template	Unstable, requires special strains, or lysate treatment	Stable
Peptide synthesis	Unstable, requires special strains, or lysate treatment	Stable
Applications	Small scale protein expression	Peptide expression for mRNA display
	Large scale protein biomanufacturing	Isotopically labeled peptides (MS standard)
	Biological process prototyping	Translation factor characterization
Compatibility with selection systems	Ribosome display	mRNA display
	Microbead display (*in vitro* compartmentation)	cDNA display
Amber suppression	Frequently used	Seldom used
Initiation reassignment	Seldom used Limited amino acid structures	Commonly used A wide range of ncAAs (Figure 5)
Elongator codon reassignment	Less used	Commonly used (residue-specific manner) A wide range of ncAAs (Figure 6)

## 2. Types of *Escherichia coli* CFPS

### 2.1. Crude Extract CFPS

The gene transcription and translation can take place in crude *E. coli* extract supplemented with rNTPs, amino acids, energy sources, and RNA polymerases. The first report of an *E. coli* crude cell-free translation system dates back to the 1960s when it was used to decipher the genetic code (Nirenberg and Matthaei, 1961; Nirenberg and Leder, 1964). Since then, many variants of the crude CFPS have been developed (Dopp B. J. L. et al., 2019) and used for elucidation of many biochemical processes, prototyping of metabolic and gene expression circuits including the glycosylation pathway, and for high yield therapeutic biomanufacturing (Carlson et al., 2012; Guarino and DeLisa, 2012; Quast et al., 2015; Jaroentomeechai et al., 2018; Kightlinger et al., 2019; Silverman et al., 2019).

Preparation of the most widely used S30 cell extract for CFPS is simple and inexpensive. It involves cell disruption followed by a run-off incubation procedure that frees the ribosomes and degrades the mRNAs, several high-speed centrifugations that removes non-essential proteins. Approximately 500–1,000 proteins were identified in *E. coli* S30 lysate (A19 and BL21 Rosetta2) (Foshag et al., 2018; Garenne et al., 2019). These represent 20–40% of the *E. coli* proteome (Iwasaki et al., 2010; Schmidt et al., 2016). The extract also contains residual amounts of membrane proteins including the respiratory chain involving several subunits of the ATP-synthase and NADH-quinone oxidoreductase. These proteins enable oxidative phosphorylation in the cell free system for energy regeneration under the condition known as “Cytomim” (Jewett and Swartz, 2004; Zawada et al., 2011; Cai et al., 2015).

The Cytomim system closely mimics *E. coli* cellular metabolism and integrates complex metabolic networks to provide new sources for ATP production (Jewett and Swartz, 2004; Jewett et al., 2008). This system has been effectively scaled up from 10 μl to 100 L and used for production of disulfide bond containing cytokine proteins, achieving 0.7 mg/ml yield in a long (>10 h) batch reaction (Zawada et al., 2011). Better understanding of and control over the chemical reactions in the cell extracts dramatically enhanced the productivity and scalability of protein synthesis, thereby transforming the crude CFPS systems into useful platforms for high yield protein production and therapeutic biomanufacturing (Carlson et al., 2012). Other energy generation systems include the PANOX-SP (acronym for PEP, Amino acids, NAD, Oxalic acid, Spermidine, and Putrescine) (Jewett and Swartz, 2004; Albayrak and Swartz, 2013; Martin et al., 2018) and 3-phosphoglyceric acid/Maltose dual system (Caschera and Noireaux, 2014; Garamella et al., 2016). They also take advantage of the *E. coli* cellular metabolism to perform ATP generation for extended periods achieving model protein production yields in excess of 1 mg/ml in batch mode.

However, the S30 system contains other factors, such as nucleases, RNases, and proteases, that negatively affect the translation productivity (Foshag et al., 2018; Garenne et al., 2019). The presence of endo- and exo- nucleases causes rapid decay of linear PCR templates. In efforts to avoid time-consuming and costly plasmid amplification and purification, linear templates were utilized to prime the translation reaction using S30 lysate from dedicated *E. coli* strains including BL21 star (DE3) (Ahn et al., 2005; Shrestha et al., 2014), SL119 (Lesley et al., 1991), NMR5 (A19 ΔrecCBD::red-kan ΔendA) (Michel-Reydellet et al., 2005). These strains are deficient in endonuclease RNase E, exonuclease V, endonuclease I, and exonuclease V, respectively. Other strategies to protect linear DNA include utilization of Lambda Gam protein (Sun et al., 2014), short auxiliary double-stranded DNAs encoding χ sites that preferentially bound to RecBCD recombination machinery (Marshall et al., 2017), steric protection of the linear DNAs with 250–500 bp protective sequences (Sun et al., 2014) and circularization of the linear fragments (Dopp J. L. et al., 2019). The presence of RNases in the cell extract causes rapid RNA degradation and prevents its use for mRNA display (Liu et al., 2000). However, S30 extract from the MRE600 strain, which is deficient in ribonuclease I, is compatible for ribosome display presumably due to at least partial RNA protection by the ribosome (Hanes and Pluckthun, 1997; Yonezawa et al., 2003; Dreier and Pluckthun, 2018). Further the commercial S30 based CFPS kit has been integrated with *in vitro* bead display for binder selection (Huang et al., 2013).

Utilization of crude CFPS for ncAA incorporation, coupled with chemoenzymatic acylated tRNAs for amber or frameshift suppression, dates back nearly 40 years to the 1980s (Noren et al., 1989). This approach suffers from low protein yield due to the single turnover of the pre-charged, non-regeneratable ncAA-tRNAs and has been largely replaced by the subsequently developed orthogonal tRNA and aminoacyl-tRNA synthetase pairs (o-tRNA/aaRSs). These are defined as orthogonal translation systems (OTSs) and have been widely interfaced with the crude CFPS for high yield ncAA-protein production mainly via amber suppression (Goerke and Swartz, 2009; Hong et al., 2014a). Recent advances in using this approach for ncAA incorporation include production of antibody drug conjugates (Zimmerman et al., 2014) and phosphoproteins with single or multiple phosphoserines (Oza et al., 2015); site-specific PEGylation (Wilding et al., 2018); incorporation of ncAAs via reassigned sense codons (Cui et al., 2017, 2018) and the use of cell-extract from genome recoded bacteria with high amber suppression efficiency (Martin et al., 2018; Des Soye et al., 2019).

### 2.2. PURE CFPS

PURE (Protein synthesis Using purified Recombinant Elements) system represents a key breakthrough in cell free synthetic biology. The system was first reported in 2001 and was a result of a thorough understanding of the mechanism of prokaryotic transcription and translation, along with advancements in the isolation of functional translational machinery (Shimizu et al., 2001; Shimizu and Ueda, 2010). The system is composed of 36 individually purified protein factors combined with purified ribosomes, total tRNA mixture, and the necessary small chemical compounds and ions. The protein factors include the T7 RNA polymerase for transcription, a full set of 20 aminoacyl-tRNA synthetases for continuous tRNA aminoacylation, 10 translation factors for initiation, elongation, and termination (initiation factors IF1, IF2, IF3, elongation factors EF-Tu, EF-Ts, and EF-G, release factors RF1, RF2, RF3, and a ribosome recycling factor RRF), as well as 5 enzymes including methionyl-tRNA transformylase, creatine kinase, myokinase, nucleoside diphosphate kinase, and pyrophosphatase. The creatine phosphate: creatine kinase system is commonly used for ATP generation in PURE system and may be responsible for its relatively short productive phase.

PURE contains negligible amounts of factors that negatively affect protein translation. Low levels of nuclease, RNase, and protease activities allow the use of linear DNA or mRNA as templates for efficient polypeptide translation. Protein folding can be improved by addition of appropriate molecular chaperons as well as by adjusting the redox potential of the reaction (Shimizu et al., 2005). This system has been used to dissect the functional mechanism of different components of protein biosynthetic machinery such as ribosome rescue factors (Shimizu, 2012) and protein chaperones (Niwa et al., 2012). It has also been used to produce membrane proteins (Kuruma and Ueda, 2015) as well as isotope-labeled peptides as mass spectrometry standards (Narumi et al., 2016, 2018).

PURE system provides superior control over individual components that can be manipulated to achieve the desired translational outcomes. Majority of the work using PURE has focused on reprogramming sense codons by omitting the corresponding amino acids and aaRSs. Development of Flexizyme (*Flexi*ble tRNA acylation Ribo*zyme*) transformed the PURE system into a very powerful platform for production of non-standard peptides. In particular it enables the production of peptides cyclized in various ways including backbone cyclisation, backbone-side chain cyclisation, as well as producing bicyclic and tricyclic peptides (Ito et al., 2013; Passioura and Suga, 2014; Bashiruddin et al., 2015; Yin et al., 2019). More than 300 different ncAAs including N-methyl and D-amino acids were incorporated into such peptides endowing them with drug-like properties (**Figures 5**, **6**).

Integration of the PURE system with Flexizyme tRNA aminoacylation technology and mRNA display has resulted in a powerful *in vitro* selection platform for constrained peptides (Passioura and Suga, 2017; Huang et al., 2019). The platform can generate macrocyclic peptide libraries with diversity approaching 10^13^. Screening of such libraries consistently yields a range of highly selective and potent protein binders capable of stabilizing protein conformations and disrupting protein: protein interactions. Such peptides can be developed as research agents, affinity ligands, vaccines as well as pharmaceutical drugs (Passioura, 2019). Recent examples include HiP-8, a macrocyclic thioether peptide consisting of 12 amino acids, that selectively recognizes active hepatocyte growth factor and can be used in potential diagnosis and treatment of cancers (Sakai et al., 2019). Another example is ub4ix that tightly and specifically binds to K48-linked Ubiquitin chains and protects them from deubiquitinating enzymes and degradation (Nawatha et al., 2019). Additionally, this macrocyclic peptide can enter cells, inhibit growth, and induce programmed cell death, opening new opportunities for therapeutic intervention (Nawatha et al., 2019).

### 2.3. Comparison of the S30 and PURE Systems

A comparison of the S30 and PURE systems is provided in Table 1. Crude CFPS is generally more economical and efficient in terms of protein production. One study demonstrated that the S30 system generated 5 times more proteins than PURE (190 and 41 μg/ml of *p*-propargyloxy-L-phenylalanine-sfGFP), and is much more cost-effective (<$0.05/reaction and $10/reaction) (Hong et al., 2014b). Recently reported variants of the S30 system produced ncAA-containing proteins at mg/ml quantities with >95% fidelity using o-tRNA/aaRS (Martin et al., 2018). However, peptides are generally unstable in S30 cell extract, which requires additional treatment to inhibit protease activity and improve product stability (Cui et al., 2015). In contrast, PURE is well-suited for peptide production due to the lack of contaminating proteases (Pardee et al., 2016).

Currently, genetic code expansion or reprogramming in crude CFPS is far less advanced than that in the PURE CFPS system as well as in the *in vivo* system. Only a limited number of ncAAs has been explored (Quast et al., 2015). Manipulation of the translational machinery, the tRNAs, aaRSs, and ribosomes, in crude CFPS is less straightforward as in the PURE system. However, it is possible to separate the native tRNA pool and the ribosome from S30 cell extract. Ablation/inactivation of a specific tRNA can liberate the corresponding sense codon(s) for coding ncAAs (Cui et al., 2018). The cell extract centrifuged at 150,000 g (S150) can be separated from the ribosomes and used for *in vitro* construction of modified ribosomes with specialized activity (Jewett et al., 2013).

## 3. Genetic Encoding of ncAAs

**Figure 1:**
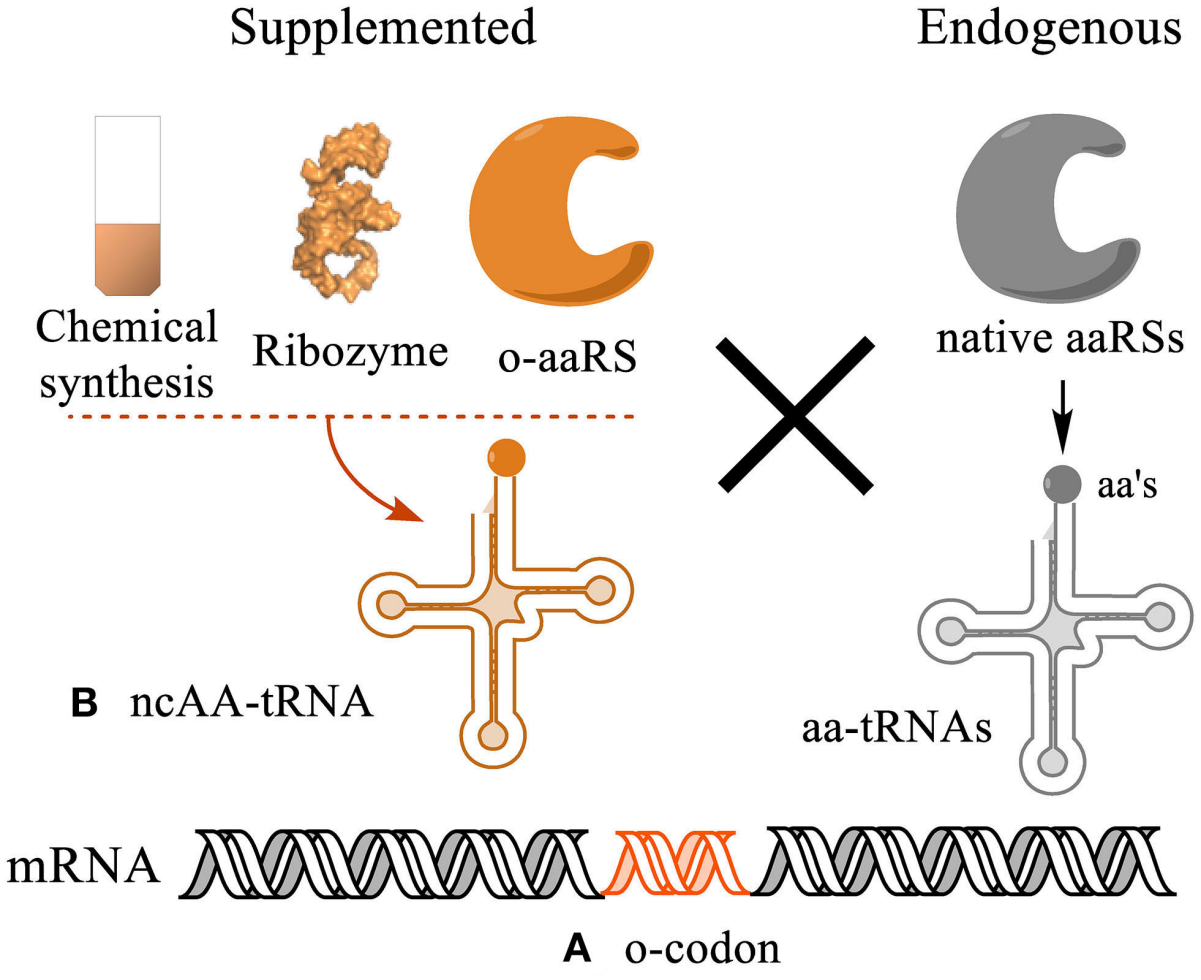
Strategies for genetic encoding of ncAAs in protein sequences. **(A)** A genetic codon needs to be liberated from decoding by endogenous tRNA/aaRS pairs to be orthogonal to the system. **(B)** Such codon is then reassigned to a ncAA using an orthogonal acylated tRNA conjugate (ncAA-tRNA). The ncAA-tRNA can be synthesized *in vitro* using chemical or chemoenzymatic synthesis, or co-translationally charged by an orthogonal aminoacyl-tRNA synthetase (o-aaRS). The efficiency and orthogonality of the ncAA-tRNA in the translation system determine the yield and homogeneity of the translated proteins.

The genetic code determines translation of 64 nucleotide triplets into 20 canonical amino acids. To incorporate ncAAs into protein sequences, a genetic codon needs to be reassigned to a ncAA by an orthogonal acylated tRNA (Figure 1). This process includes two key elements: (1) Creation of orthogonal codons decoded only by orthogonal acylated tRNAs but not by any endogenous acylated tRNAs. (2) Synthesis of orthogonal ncAA-tRNA conjugates that specifically recognize the orthogonal codon, but not the other codons. This can be carried out both *in vivo* and *in vitro* in two fashions: site specific and residue specific.

### 3.1. Creation of Vacant Codons for Reassignment

The first prerequisite for ncAA incorporation is creation of an orthogonal codon that is not decoded by the endogenous tRNA/aaRS pair(s). This could be natural triplets (stop and sense codons), unnatural triplets, as well as four-base-codons i.e., Quadruplets (Table 2). Incorporation of ncAAs via reassignment of stop or quadruplet codons results in site-specific incorporation. Sense codon reassignment can be performed in both site- and residue-specific fashion depending on the orthogonality generation approach (Table 2). Site-specific sense codon reassignment means the reassignment of a subset of synonymous sense codons (mostly one or two) to a ncAA while the rest of synonymous codons are retained for the natural amino acid, leading to genetic code expansion. Residue-specific approach indicates the reassignment of all the synonymous codons of a natural amino acid to a ncAA (genetic code reprogramming). This will be discussed later in detail.

**Table 2:** Choice and Method for creating Orthogonal Codons.

**Potential o-codons**	**Number**	**Orthogonal**	**Orthogonality creation**	**Competition**	**Reassignment mode**
Stop codons	3	Yes	—	RF1	Site-specific
Sense codons	61	No	Deletion of a specific aa ± aaRS	aa-tRNA(s)	Residue-specific
		No	Deletion of the cognate tRNA(s)	aa-tRNA(s)	Site-specific
Quadruplets	256	Yes	—	aa-tRNA(s) that recognize the 1st three bases as triplets	Site-specific
Triplets with novel base(s)	variable	Yes	—	Depending on the orthogonality of new bases	Site-specific

#### 3.1.1. Stop Codon and Nonsense Suppression

**Figure 2:**
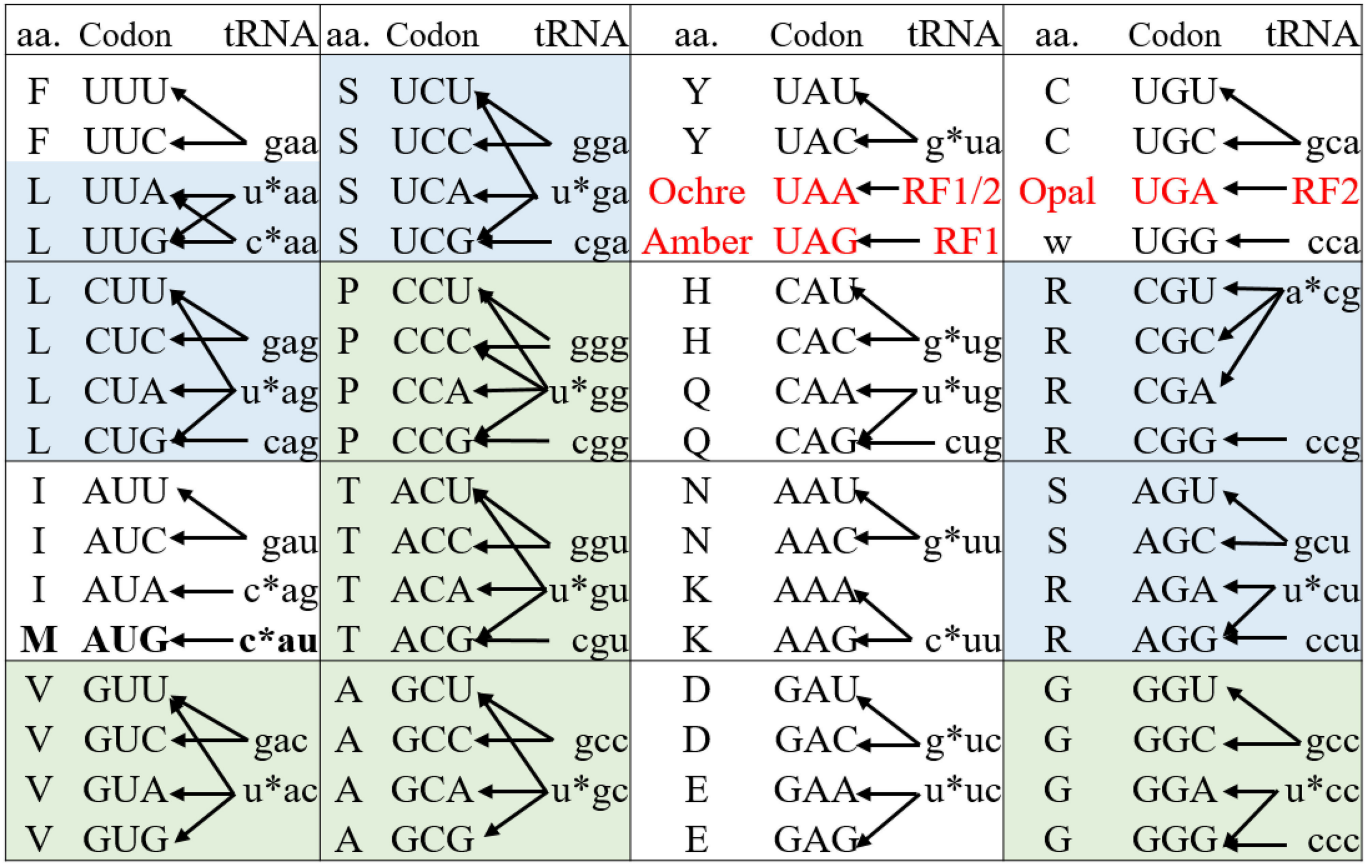
The genetic code and its decoding tRNA isoacceptors of *E. coli*. The genetic codons are shown in upper case while their decoding tRNAs are shown in lower case representing by their respective anticodons. The * indicates the modification on the nucleotide at 34 position of the native tRNAs (N_34_). The three stop codons (red) are read by release factors to signal the translation termination. The 6- and 4-fold degenerate amino acids that can be used for sense codon reassignment are highlighted in blue and green, respectively. The Met AUG codon (in bold) acts as initiator and elongator via decoding by fMet-tRNA^iMet^CAU and Met-tRNA^Met^CAU, respectively. These two acylated tRNAs are delivered to the ribosomal P site by IF2 and to A site by EF-Tu for translation initiation and elongation, respectively.

Nonsense codons (*amber, opal*, and *ochre*) that do not have decoding tRNA/aaRS pairs, are naturally orthogonal to both *pro-* and *eukaryotic* protein synthesis systems (living organisms and CFPSs). Protein translation generally terminates at these codons through the action of release factors (Figure 2). Amber codon is the least used stop codon in the *E. coli* genome (frequency of 7–9%) and is the most used codon for site-specific ncAA incorporation. Many approaches have been developed to increase the *amber* suppression efficiency by silencing or removing competing RF1 from the system (Martin et al., 2018; Adachi et al., 2019). *Ochre* codon that faces competition from both RF1 and RF2 is less frequently used for genetic code expansion (Wan et al., 2010; Odoi et al., 2013). Background suppression of *opal* codon is generally high (10–25% of the wild type protein activity), leading to contamination of the modified proteins with mis-incorporated Trp (O'Donoghue et al., 2012; Odoi et al., 2013). This is likely to occur due to a near-cognate suppression of UGA codon by tRNA^Trp^CCA through wobble base pairing at the 3rd codon position.

Nonsense suppression generally limits the number of incorporable ncAAs types to two, as one stop codon needs to be reserved for translational termination. However, a recent publication demonstrated the simultaneous utilization of all the three nonsense codons to encode three distinct ncAAs (Italia et al., 2019).

#### 3.1.2. Sense Codons and Sense Codon Reassignment

The key obstacle in the reassignment of sense codons is the presence of aminoacyl-tRNAs (aa-tRNAs) that compete with the introduced ncAA-tRNA conjugates (Figure 2). Depending on the approach used to prevent the interference of native acylated tRNAs, sense codon could be reassigned either in residue- or site-specific fashion.

In principle eliminating certain amino acid from the translation reaction can free all its synonymous codons leading to reassignment of all triplets encoding for that amino acid. However, in reality it is very difficult to completely remove residual amount of that amino acid from the system. A notable exception is the AUG start codon which could be faithfully assigned to a ncAA by simply omitting Met in the translation reaction. In the PURE system, simultaneous exclusion of both the amino acid and the corresponding aaRS is effectively used to liberate the codons (Goto et al., 2011). All the synonymous codons for the same amino acid are reassigned to a ncAA. This approach is referred to as “genetic code reprogramming” since the genetic code is recoded from one canonical amino acid to a ncAA in residue-specific manner (Ohta et al., 2007).

In the crude CFPS, S30 lysate prepared from an auxotroph strain or treated by gel filtration was used to deplete certain amino acid from the system (Torizawa et al., 2004; Singh-Blom et al., 2014). By taking advantage of the promiscuity of certain aaRS, this approach was adapted for *in vitro* synthesis of streptavidin protein with tryptophan analogs (Singh-Blom et al., 2014). However, drastic change of protein composition in residue-specific manner from one canonical amino acid to a ncAA is sometimes detrimental to protein functionality and limits this approach to applications such probing proteomic changes in specific cell types (Saleh et al., 2019).

In contrast, to achieve site-specific ncAA incorporation using sense codon reassignment the degeneracy of the genetic code should be broken to liberate at least one codon for ncAA incorporation while maintaining the decoding of other synonymous codon(s) to the native amino acid. In the *E. coli* genome, more than 40 tRNAs decode the 61 sense codons to 20 amino acids (Figure 2) (Cui et al., 2015). The decoding preference provides a valuable guide for identifying “orthogonal” vs. “native” codon pairs from the synonymous codons of a particular amino acid. Such pairs can either be created from the codons of different families of 6-fold-degenerate amino acids or from those derived from the unsplit codon family boxes with restricted wobble recognitions. The cognate tRNA(s) should be inactivated or excluded from the translation system to create orthogonal codons.

The Suga group replaced the native tRNA mixture with 32 synthetic tRNAs in the PURE system (Iwane et al., 2016). This resulted in genetic code expansion to ncAAs while maintaining 20 canonical amino acids. They demonstrated the expansion of the amino acid repertoire from 20 to 23 by artificially dividing the upsplit codon boxes of Arg, Gly, and Val where the G- and C-ending codons in the same codon boxes were translated to 2 amino acids (1 canonical amino acid +1 ncAA) using wild type synthetic tRNASNN/aaRS pair and precharged ncAA-tRNA^AsnE2^SNN (S=G or C), respectively. The drawback of this approach is in its complexity, and in the effort required to synthesize and purify the system's individual components including synthetic tRNAs.

In an alternative approach our group created S30 extract chromatographically depleted of all tRNAs. The translational activity of such cell extract is dependent on the supplementation with a semi-synthetic tRNA complement (Cui et al., 2015). Similar to Suga's method, the requirement to synthesize the semi-synthetic tRNA complement complicates broad utilization of the method. We circumvented this problem by utilizing DNA-hybridization chromatography to deplete the native tRNA mixture of certain tRNA isoacceptors thereby creating vacant codons (Cui et al., 2017). In a further development of this approach we bypassed chromatography steps by sequestering specific tRNA(s) in lysate using methylated antisense oligonucleotides (Cui et al., 2018). These form essentially irreversible complex with the desired tRNA(s) sequestering them from the translational machinery. Using this approach, we demonstrated reassignment of AGC/U and AGG codon to ncAAs while retaining UCN and CGN for Ser and Arg, respectively. This approach is simple and scalable as it involves only liquid handling. Furthermore, it is applicable to cell-free systems of any origin. We demonstrated this by applying the approach to a *Leishmania* derived eukaryotic cell-free expression system (Cui et al., 2018).

#### 3.1.3. Quadruplet Codons and Frameshift Suppression

Another approach for site-specific ncAA incorporation is frameshift suppression. This approach was applied to crude CFPS in conjunction with chemoenzymatic synthesized ncAA-tRNAs (Quast et al., 2015). Theoretically quadruplet codons could provide 256 blank codons. However, encoding a ncAA via four-base codons is far less efficient than the use of nonsense suppression, whereby the yield of a target protein is lowered by misreading of the quadruplet as a triplet. The most commonly used quadruplets *in vivo* comprise rare triplets plus a fourth nucleotide, such as AGGN (Neumann et al., 2010; O'Donoghue et al., 2012). Use of rare codons is preferred to reduce the competition with endogenous acylated tRNA(s). Modified ribosomes were used to enhance the quadruplet's suppression efficiency (Neumann et al., 2010).

#### 3.1.4. New Base Pairing

Unnatural base pairs (UBPs) hold the promise to dramatically increase the information storage of DNA, RNA, and therein encoded protein sequences. However, retrieval of the genetic information stored in the DNA molecules requires a series of biochemical steps ranging from faithful DNA replication, transcription of the UBPs in the context of the DNA templates to RNAs (mRNAs and tRNAs), and finally efficient aminoacylation of tRNAs harboring the modified anticodons that are capable of decoding the triplets with UBPs in the ribosomal center. Ensuring that all these steps proceed with sufficient affinities and fidelity requires a large engineering effort.

**Figure 3:**
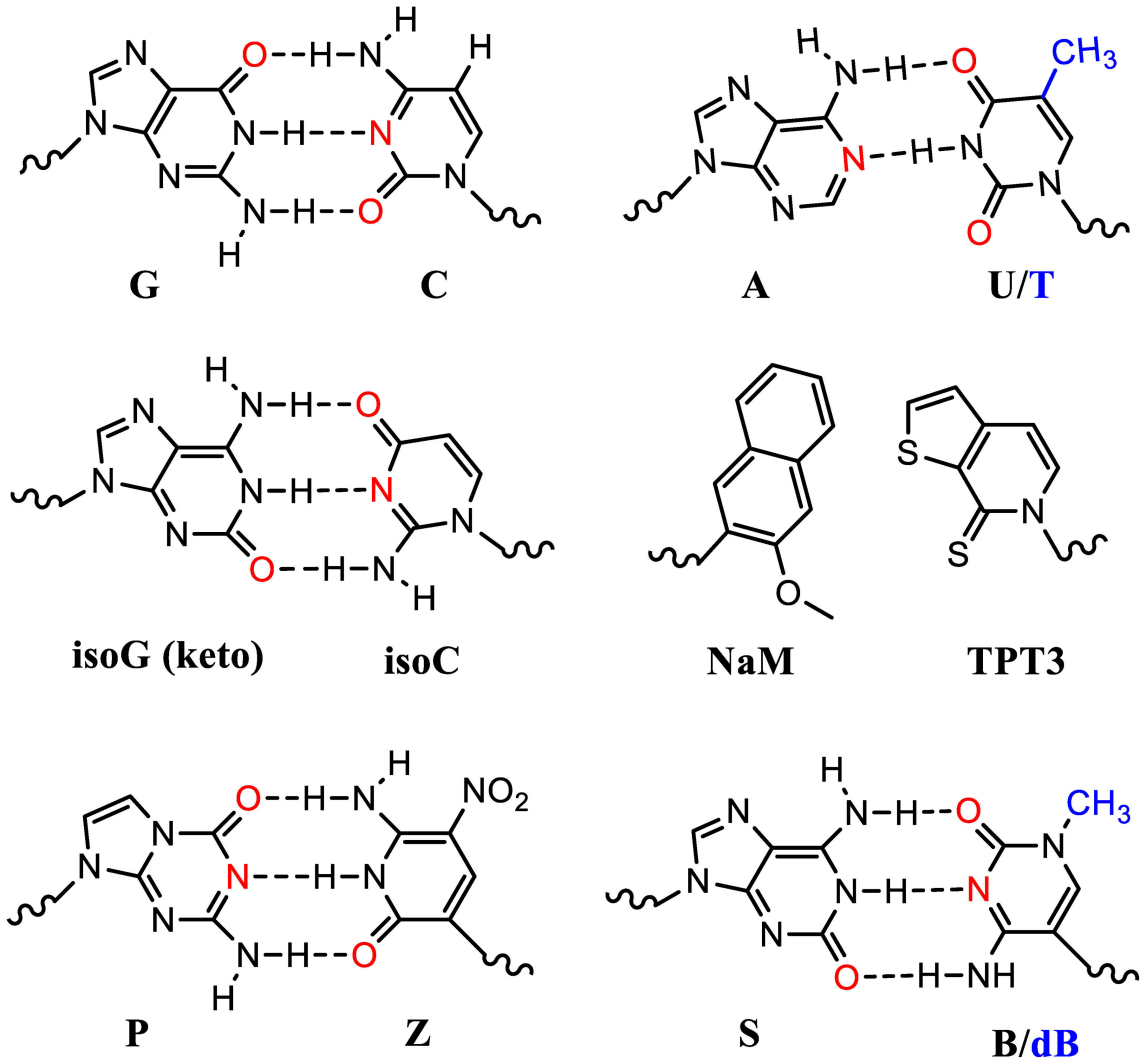
Standard and unnatural base pairs (UBPs). Nature has evolved 5 nucleotides (ATGCU) in natural genetic sequences (DNA and RNA). The sugar and phosphate groups are omitted here for simplicity. Many UBPs are developed, based on either hydrophobic interactions or hydrogen bonding. The electron donors in the bases are highlighted in red. The T and dB in DNA molecules contain an extra methyl group (blue) compared to U and B in the corresponding RNA molecules.

Many UBP pairs have been reported (Malyshev and Romesberg, 2015). These include isoG (keto): isoC pair which mimics the natural G:C pair forming 3 hydrogen bonding (Figure 3). The ribosomal decoding of the unnatural codons (*iosC*AG) using a tRNA harboring its complementary anticodon (CU*isoG*) is effective in mediating ncAA incorporation (Bain et al., 1992). However, isoG could isomerize and form hydrogen bonding with T, which diminishes its orthogonality causing elimination after several rounds of DNA replications.

UBPs based on hydrophobic interactions such as dNaM-dTPT3 in DNA molecules have been used to build semisynthetic organism (SSO) (Zhang Y. et al., 2017; Fischer et al., 2020). DNA templates with these UBPs can be transcribed into mRNA and tRNA molecules with the respective unnatural nucleotides containing TPT3 and NaM (Figure 3). These novel tRNAs could decode the unnatural codons to ncAAs using o-aaRSs. Theoretically, one extra UBP pair could expend the genetic code from 64 to 216 triplets. However, some UBP-containing codons suffer with poor *in vivo* retention and require further optimization (Dien et al., 2018). Fischer et al. recently systematically analyzed a range of unnatural codons containing this UBP pair and identified at least nine codons capable of mediating protein production with little or no detectable contamination with native proteins (Fischer et al., 2020). Impressively, they demonstrated that at least 3 of these unnatural codons were mutually orthogonal and could be simultaneously used to decode three distinct amino acids including two ncAAs. This study showcased the first 67-codon organism with UBPs demonstrating a possibility of creating stable SSO with chemical metabolism significantly different from native organisms. Of note is that the lack of interbase hydrogen bonding in these UBPs restricts their usage to the 2nd or 3rd position of the codons. Poor decoding of the unnatural triplets with a UBP at the 1st position is possibly due to their failure to adopt an appropriate structure or to engage the type I A-minor interaction, which is crucial for selecting correct Watson-Crick like geometry at the ribosomal decoding center.

Another two nucleotide analog pairs interacting via hydrogen bonding (P:Z, B:S) were recently reported increasing the number of units in the genetic alphabet from 4 to 8 (A, T, G, and C, purine analogs P and B, and pyrimidine analogs Z and S) (Hoshika et al., 2019). The P:Z and S:B (or S:dB in RNA) pairs are promising UBPs that mimic the natural nucleotides and are compatible with DNA replication and RNA transcription (Figure 3). With increased information density and storage capacity, this 8-letter DNA/RNA genetic system holds the promise of dramatically increasing diversity of building blocks for protein synthesis. However, the efficiency and orthogonality of these UBPs compared to the natural ones in terms of codon decoding in the ribosomal center still need to be evaluated.

### 3.2. Orthogonal ncAA-tRNAs

Acylation of an orthogonal tRNA with the desired ncAA to generate ncAA-tRNA conjugate is another key step in genetic code expansion and reprogramming. Numerous synthetic routes have been developed, including the aforementioned chemoenzymatic tRNA acylation (Hecht et al., 1978), enzymatic acylation with orthogonal aaRSs (Wang et al., 2006b) or ribozymes such as Flexizyme (Murakami et al., 2006), as well as some other less used approaches which take advantage of the promiscuity of native aaRSs (Hartman et al., 2006, 2007; Iqbal et al., 2018) and post-aminoacylation modifications (Merryman and Green, 2004; Gubbens et al., 2010). Besides the aaRS route, the majority of the acylation methods generate ncAA-tRNA conjugates *in vitro* and are supplemented into the translation reaction where they support single turnover translation thereby limiting the yield of modified polypeptides.

Chemoenzymatic and Flexizyme acylation are among the most used *in vitro* tRNA acylation methods, enabling the incorporation of chemical functionalities that are quite distinct from native amino acids and therefore cannot be conjugated via aaRS route. The former involves enzymatic ligation of chemically prepared acylated dinucleotides to truncated tRNAs lacking the 3'-CA dinucleotide (Hecht et al., 1978). This, well-established, method is theoretically applicable to any substrates but requires multiple steps of chemical synthesis and suffers from low yield. The Flexizyme represents an important advancement in tRNA acylation allowing incorporation of a wide range of ncAAs into polypeptides.

A recent exciting work on directed evolution of ribozymes identified the so-called T-boxzyme, which enabled *in situ* aminoacylation of tRNA^Gly^GCC and one-pot synthesis of peptides with N-terminal biotin group (Ishida et al., 2020). Unlike the Flexizyme, this T-boxzyme is based on the T-box riboswitch of *Bacillus subtilis* glyQS and thereby is tRNA^Gly^ specific. Although co-translational charging of ncAA via T-boxzyme was showcased, the efficiency of peptide synthesis was several times lower than that using supplemented ncAA-tRNA conjugate. Further optimization is expected to turn this technology into a useful tool.

#### 3.2.1. Flexizyme Approach

##### 3.2.1.1. Flexizyme and its substrates

**Figure 4:**
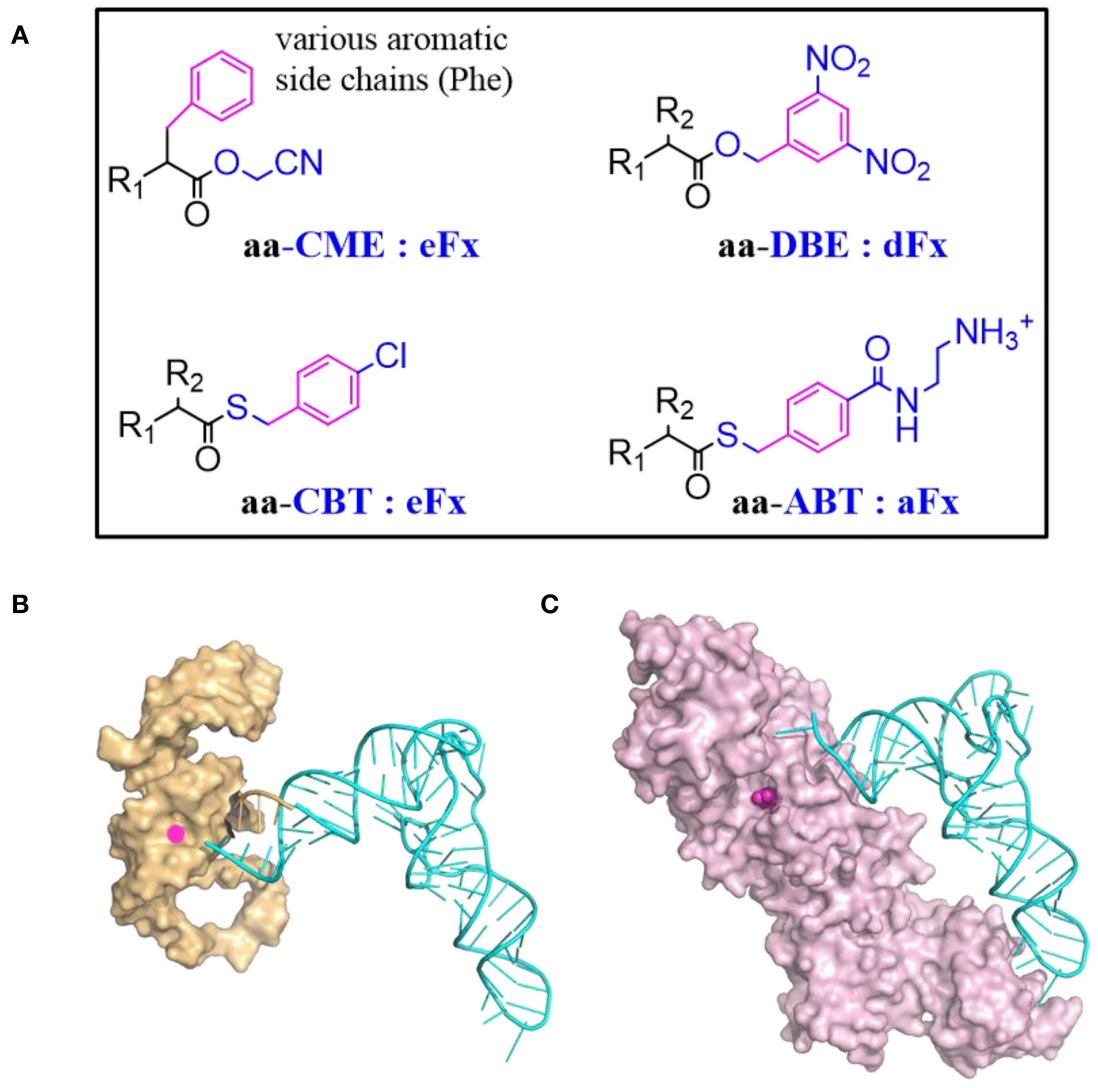
tRNA acylation via Flexizyme and o-aaRS approach. **(A)** The choice of amino acid activation group and the charging Flexizyme (Fx). The Fx can charge a range of substrates including amino acids, hydroxyl acids, N-acyl amino acids etc. R_1_ = NH_2_, OH, RCONH, etc; R_2_ = various side chains. The acid substrates are colored in black while the activation or leaving group is colored in blue. The aromatic moiety in the substrate that recognized by Fx is highlighted in magenta. Aromatic amino acids are activated by cyanomethyl ester (CME) and charged on tRNAs via eFx while non-aromatic amino acids are activated by 5-dinitrobenzyl ester (DBE) and charged on tRNAs via dFx. For acyl-donor substrates with α-N-acyl, β-branched, or bulky side chains, the best choice is eFx paired with the 4-chlorobenyl thioester (CBT) leaving group. If the activated acids with above leaving groups are of poor solubility, pairing with aFx and 4-[(2-aminoethyl) carbamoyl] benzyl (ABT) thioester leaving group might be a better choice. **(B)** A representative structure of Fx and tRNA complex. Fx (PDB: 3CUN) recognizes the tRNA through its 3'-terminal region (5'-D*CCA*-3' where D means A/G/U) therefore it accepts most of tRNAs regardless of their body sequences. The “GGU” end of Flexizyme is shown as cartoon in orange to highlight its complementary interaction with the “CCA” end of tRNA (Cyan). **(C)** A representative structure of tRNA and aaRS complex (MjtRNA^Tyr^/TyrRS, PDB: 1J1U). The amino acid binding pocket is filled with tyrosine (magenta). Most of aaRSs recognize tRNAs through the two distal extremes involving the anticodon sequence and the N_73_ discriminator base. The substrate binding is malleable but limited to analogs of canonical amino acids.

Flexizyme was created to mimic the function of aaRSs but using RNA as catalyst (Morimoto et al., 2011). A Flexizyme family includes dinitro-flexizyme (dFx) (Murakami et al., 2006), enhanced flexizyme (eFx) (Murakami et al., 2006), and amino-flexizyme (aFx) (Niwa et al., 2009). These ~45 nucleotide long RNA molecules can acylate tRNAs using a wide range of substrates with appropriated leaving groups (Figure 4A). Acylation efficiency varies depending on the structure of the acyl-donor substrate, the leaving group, and the type of flexizyme (Goto et al., 2011).

Recently, a large panel of substrates including Phe analogs, benzoic acid derivatives, heteroaromatic amino acids, and aliphatic acids were synthesized to study the Fx acceptance rules (Lee J. et al., 2019). Reaction rates of eFx toward its aromatic substrates generally correlate with the electronic character of the substrate benzol ring, increasing for electron-poor substrates while decreasing for electron-rich substrates. The effective substrates could form either T-stacked or parallel stacked interactions with eFx. The aFx prefers aliphatic substrates with straight chain, while the substrates with increased steric bulk decrease aFx-catalyzed acylation activity.

##### 3.2.1.2. tRNAs engineering for Flexizyme mediated ncAA incorporation

The Flexizyme theoretically can recognize any tRNA with 3'-*D*_73_*C*_74_*C*_75_A_76_, regardless of their body sequences (Figure 4B). An engineered tRNA^fMetE^, derived from *E. coli* initiator tRNA with a single mutation (C1G), is commonly used for reassignment of the initiation codon (Goto et al., 2011). This tRNA harbors elements that interact with many translational factors (IF2 and IF3) to initiate the translation (Rasmussen et al., 2009). The C1G mutation increases T7 transcription efficiency and thereby the yield of this synthetic tRNA.

Engineering elongator tRNAs is a common practice to enhance ncAA incorporation efficiency in CFPS, and several generations of o-tRNAs have designed. *E. coli* tRNA^AsnE2^NNN maintains orthogonality to all the 20 aaRSs and is effective in incorporating a single copy of many functional chemistries but shows little or no yield for consecutive incorporation of structurally challenging ncAAs, such as D-amino acids (Ohta et al., 2007; Goto et al., 2009, 2011). The engineered tRNA^GluE2^ displays strong EF-Tu binding and was used to achieve incorporation of consecutive D-Ser amino acids (Katoh et al., 2017a). The rationally designed tRNA^ProE2^ (**Figure 8E**) has the optimal T-stem motif from tRNA^GluE2^ for enhanced EF-Tu binding and is capable of recruiting EF-P to facilitate synthesis of peptides with challenging substrates. The tRNA^ProE2^ is effective in mediating ribosomal synthesis of polypeptides with not only consecutive D-amino acids (D-Phe, D-Ser, D-Ala, and/or D-Cys) (Katoh et al., 2017a) but also β-amino acids (Katoh and Suga, 2018) and 2-aminoisobutyric acid (**Figure 6**, compound **57-58, 59-61, and 68**) (Katoh et al., 2017b). The design of tRNA^ProE2^ is inspired by the recent breakthrough in discovery of the recognition elements in tRNA for recruiting EF-P for enhanced peptide bond formation (Katoh et al., 2016). The determinant of EF-P recognition relies on the D-loop structure (9-nt loop size) closed by the stable 4 base pairs in the D-stem (**Figure 8E**).

Noticeably, changing the codon-anticodon base paring or the whole progenitor tRNAs might affect ncAA incorporation. For instance, precharged tRNA^AsnE2^CUA with D-Cys or D-Met is not effective for amber suppression (Goto et al., 2008a) while the same D-amino acid-tRNA but with GGA anticodon is capable for reassigning UCC (Ser) codon (Fujino et al., 2013). Compared to tRNA^AsnE2^GGA that affords single-incorporation of 12 D-amino acids, an unmodified tRNA^Gly^ supports single incorporation of 17 out of 18 tested D-amino acids into a polypeptides (Achenbach et al., 2015). This might be due to the stronger EF-Tu binding affinity of tRNA^Gly^ (Asahara and Uhlenbeck, 2002). The peptide yield mediated via precharged-tRNA^GluE2^ is several times higher than that of pre-charged ncAA-tRNA^AsnE2^ (Terasaka et al., 2014).

##### 3.2.1.3. Flexizyme enabled ncAAs incorporation

**Figure 5:**
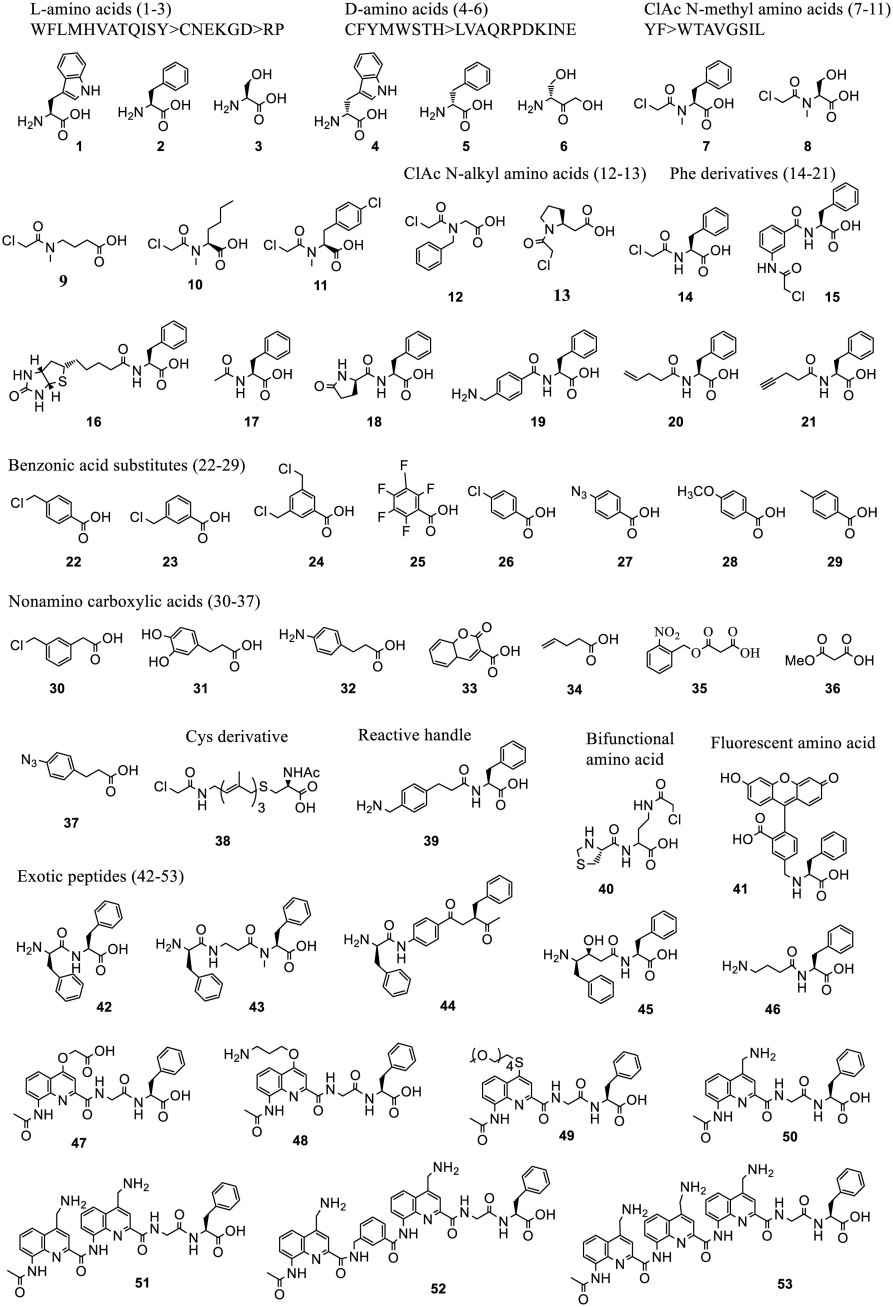
Representative acid substrates that were incorporated into polypeptides using Flexizyme acylated tRNA conjugates for reassignment of the initiation codon. Most of the tested substrates except the L-amino acids (1-3) are backbone modified. The incorporation efficiency of these substrates vary (Supplementary Table 1). In some cases, a group of substrates, including 20 L-, 19 D-amino acids, 10 ClAc N-methyl amino acids, based on the canonical aa's (labeled as single letters) were tested and divided into groups linked with “>” indicating the decrease of incorporation efficiency.

**Figure 6:**
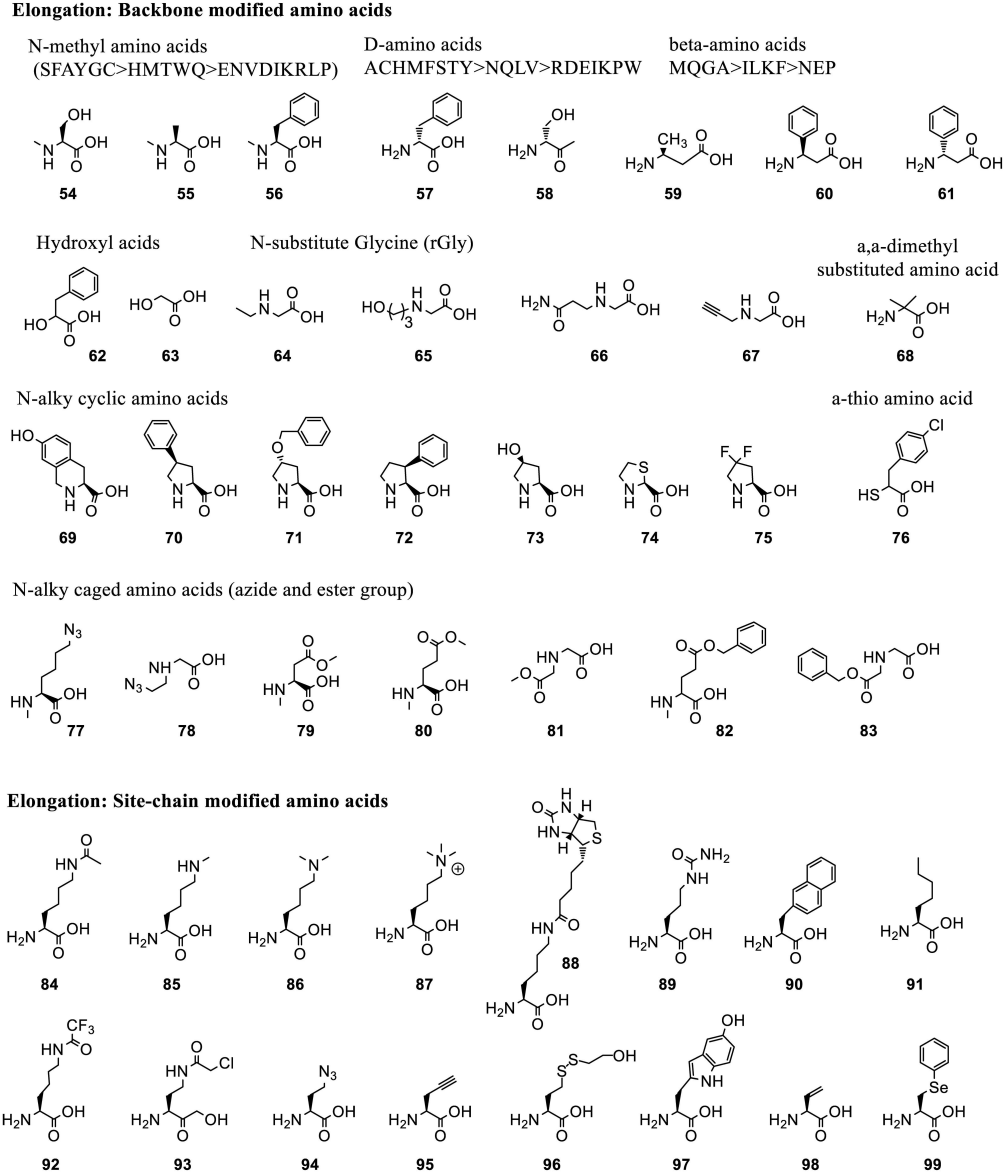
Representative ncAAs that were incorporated into polypeptides using Flexizyme acylated tRNA conjugates for elongation reassignment. These ncAAs are divided into backbone modified and side chain modified amino acids. The structure of ncAAs affects the elongation efficiency (Supplementary Table 2). In some cases, a group of substrates, including N-methylated, D-amino acids and β-amino acids, based on the canonical ones (labeled as single letter) are tested and divided into groups linked with “>” indicating the decrease of suppression efficiency.

Hundreds of acid substrates, including α-amino acids (D-, N-acyl, N-alkyl), β-amino acids, γ-amino acids, α-hydroxyl acids, α-mercapto acids, and thio acids, have been charged on tRNAs using Flexizyme and successfully installed into polypeptide sequences (Figures 5, 6). Here we review backbone- and side chain-modified ncAAs used in initiation and elongation reassignment.

*3.2.1.3.1. Initiation reassignment*. Using Flexizyme synthesized ncAA-tRNA^fMet^CAU in a Met-free PURE system, peptides with a range of ncAAs of exotic structures at N-term were synthesized with varying efficiency. A summary of these ncAAs and their incorporation efficiency are provided in Supplementary Table 1. Some of the representative structures are shown in Figure 5.

Briefly, these ncAAs include L-amino acids (**1, 2, 3**) (Goto et al., 2008b), D-amino acids (**4, 5, 6**) (Goto et al., 2008a), chloroacetyl N^α^ -alkyl amino acids (**7-13**) (Kawakami et al., 2016), Phe derivatives containing chloroacetyl- (**14-15**), biotinyl- (**16**), N-acetyl- (**17**), pyroglutamate (**18**), and other diverse groups (**19-21**) (Goto et al., 2008b; Kawakami et al., 2013a), benzoic acids derivatives (**22-29**) and other carboxylic acids (**30-37**) (Kawakami et al., 2016; Ad et al., 2019; Lee J. et al., 2019) which include aramid precursors and malonyl (1,3-dicarbonyl) substrates to generate a diverse set of aramid-peptide and polyketidepeptide hybrid molecules. Reassignment of the initiator codon has also been achieved to N-acetyl-S-12-(ClAc)farnesyl-D-Cys (**38**) (Torikai and Suga, 2014) mimicking the polyene moiety in the natural biologically active products, phenylalanine derivative with benzylamine group (**39**) for oxidative coupling, a thiazolidine-protected cysteine with 2-chloroacetoamide side chain (Thz-Cab) (**40**) for backbone cyclization (Takatsuji et al., 2019), bulky fluorescent amino acids for peptide detection and quantitation (**41**) (Terasaka et al., 2014), exotic peptides of non-standard structures containing monomers, such as D-, β-, N-methyl, aminobenzoic acids (**42-44**), γ-amino acids (**45-46**) (Goto and Suga, 2009; Ohshiro et al., 2011), as well as aromatic foldamers comprising of quinoline (Q) and pyridine (P) amino acids (**47-53**) (Rogers et al., 2018; Tsiamantas et al., 2019).

The initiator tRNAs charged with ncAAs containing N^α^-acyl groups generally afford higher peptide expression levels (Supplementary Table 1). They circumvent the formylation step by methionyl-tRNA formyltransferase (MTF) and bind to IF2 for delivery to the P-site of the ribosome. L-amino acid initiated peptides are generally formylated at their N terminus by MTF (Goto et al., 2008b) while the D-amino acid initiator is not (Goto et al., 2008a) possibly due to the steric clash of these amino acids in MTF catalytic center. The pre-acetylation of D-amino acids could enhance the translational efficiency.

Initiation reassignment to N-chloroacetyl-Phe/Trp/Tyr either in L- or D- stereochemistry integrated with downstream Cys is the most used method for generating a macrocyclic peptide library for mRNA display (Passioura and Suga, 2017; Huang et al., 2019). The N-ClAc group reacts with Cys *in situ* after translation and leads the formation of head-side chain thioether. A recently developed multiple-step strategy demonstrated the first example of backbone cyclization compatible with mRNA display (Takatsuji et al., 2019). This takes advantage of two ncAAs, the backbone modified acid (**40)** and a-thio acid (**76)**, with a downstream Cys. The puromycin molecule at the C-termini of the peptide is maintained after backbone cyclization preserving the genotype and phenotype linkage.

*3.2.1.3.2. Elongation reassignment to backbone modified residues*. Many backbone-modified residues are compatible with ribosomal elongation, such as N-methyl amino acids (**54-56**) (Kawakami et al., 2008), D-amino acids (**57-58**) (Fujino et al., 2013), β-amino acids (**59-60**), D-β-amino acids (**61**) (Fujino et al., 2016), α-hydroxyl acids (**62-63**) (Ohta et al., 2007), N-substituted Gly (rGly, **64-67**) (Kawakami et al., 2008), α, α-dimethyl substituted amino acids (**68**) (Katoh et al., 2017b), cyclic N-alkyl amino acids (**69-75**) (Kawakami et al., 2013a), α-Thio acids (**76**) (Takatsuji et al., 2019), and N-alkyl amino acids with caged side-chains (**77-83**) (Kawakami et al., 2014) (Figure 6). The incorporation efficiency is affected by the properties of the backbone as well as of the side chains (Supplementary Table 2). Generally, substrates with bulky and charged side chains are unfavorable for elongation.

Polyesters with up to 12 α-hydroxyl acids including phenyllactic acid (**62**), non-aromatic *a*-hydroxy (**63**) and their derivatives can be synthesized (Ohta et al., 2007). Up to 10 N-methyl amino acids with aromatic or non-charged and non-bulky side chains could be incorporated into the peptide at around 10–20% suppression efficiency (Kawakami et al., 2008). N-substituents of rGly bearing non-branched alkyl chain, or with no bulky group near the amino group and uncharged functional group are well-incorporated in peptoids synthesis (Kawakami et al., 2008). The expression level of peptoids decreases with the increasing number of rGly in the sequence. This is less efficient than N-methyl-peptidyl elongation with the same number of monomers. D- and β-amino acids are also compatible with the ribosomal synthesis when combined with appropriately engineered tRNAs (Fujino et al., 2013, 2016). N-alkyl amino acids with charged side chains, such as amine and carboxyl group, are poor substrates for ribosomal synthesis. This issue is circumvented by masking the charge of the side chains with precursor amino acids, azide and ester (**77-83**), followed by their chemical or enzymatic conversion back to the original group (Kawakami et al., 2014).

*3.2.1.3.3. Elongation reassignment to side chain modified residues*. Many ncAAs with side chain modifications are incorporated into peptide via Flexizyme-mediated approach (Figure 6). Lysine analogs including ε-N-acylation, ε-N-methylation (mono-, di-, or trimethylation) (**84-87**) (Kang et al., 2008) were used to study the role of post-translational modifications in protein-protein interaction and cell signaling. A lysine derivative with biotin group (**88**) was used as an affinity handle. The ncAAs with varying hydrophobicity and properties (**89-91**) (Passioura et al., 2018) were used to build libraries with expanded diversity for peptide selection (Kawakami et al., 2013b). A warhead ncAA, ε-N-trifluoroacetyl-Lysine (**92**), in peptide sequence was used to achieve selective inhibition of the de-acylation activity of Human Deacetylase SIRT2 (Morimoto et al., 2012).

Many side chain modified ncAAs are employed to form intermolecular bonds, such as N^γ^-(2-chloroacetyl)-α, γ-diaminobutylic acid **(**Cab, **93)** that reacts with Cys (Sako et al., 2008a), γ-azidohomoalanine (Aha, **94**) that reacts with propargylglycine (**95**) (Sako et al., 2008b), benzylamine in F^bza^ (**39**) that reacts with 5-hydroxytryptophan (W^OH^, **97**) in the presence of potassium ferricyanide K_3_Fe(CN)_6_ (Yamagishi et al., 2009). Other ncAAs are used to indirectly form intermolecular interactions or mimic the natural bioactive products, such as backbone cyclization of linear peptides bearing a C-terminal Cys-Pro-Glycolic acid (**63**) sequence with an N-terminal amino group (Kawakami et al., 2009) or with γ-amino acids (Ohshiro et al., 2011). Synthesis of methyllantionine-containing cyclic peptides takes advantage of vinylglycine (**98**) (Goto et al., 2009). Synthesis of alkylamides uses Aha (**94**) or 2-mercaptoethanol-masking homocysteine (**96**), and phenyllactic acid (**62**) (Nakajima et al., 2009), while synthesis of thiopeptide scaffolds utilizes Se-phenylselenocysteine (**99**) (Fleming et al., 2019).

*3.2.1.3.4. Comparison of structural acceptance in translation initiation and elongation*. The presence of an N-acyl group in ncAA positively affects its initiation but not elongation. The higher structural tolerance of ncAAs as initiators rather than elongators might be due to the relaxed recognition pattern in the ribosomal P-site. The larger substrate binding pocket in P site allows installation of not only bulky amino acids but also exotic peptides with variable chemistries in the monomer, such as N-methyl, D-, D-methyl, γ-amino acids, and foldamers (Figure 5). These substrates can not serve as elongators as they do not fit in the ribosomal A-site where precise positioning of ncAA-tRNA is critical for nucleophilic attack of the α-amino group of elongator aa-tRNA on the carbonyl group of the initiator or peptidyl-tRNAs. However, elongation reassignment can mediate multiple ncAAs incorporation while the use of the initiator codon results in a single incorporation.

#### 3.2.2. O-tRNA/aaRS Approach

Engineering o-tRNA/aaRS pairs, which do not recognize any endogenous amino acids and tRNAs but specifically charge o-tRNAs with ncAAs, has revolutionized the genetic code expansion field (Wang et al., 2006b). Although the incorporable chemistries are generally limited to amino acid side-chain analogs, this approach requires fewer chemical manipulation steps, is applicable to both *in vivo* and *in vitro* translation systems and enables co-translational charging with multiple turnovers and higher protein yields. While majority of the o-tRNA/aaRS pairs were developed in living organisms using multiple rounds of negative and positive selections, some of them have been expressed in *E. coli* strains that were used to generate cell extracts (Oza et al., 2015) or supplemented as purified components into the CFPS (Cui et al., 2018) for producing ncAA(s)-proteins. Here we provide an overview of available OTSs developed to use in *E. coli*. Although some of them, at present, were only investigated in cells, they are useful elements as ncAA encoding toolbox for more diverse applications of CFPS.

##### 3.2.2.1. O-tRNA/aaRS pairs

Two o-tRNA/aaRS pairs, *Methanocaldococcus jannaschii* (Mj) tRNA^Tyr^/TyrRS and *Methanosarcina* tRNA^Pyl^/PylRS, that have minimal or no cross-aminoacylation in *E. coli* have become the most commonly used starting points for directed evolution aimed at creation of specificity to ncAAs (Wang et al., 2006b; Liu and Schultz, 2010; Dumas et al., 2015). Mutants from these two enzymes account for two thirds of the ~200 ncAAs that were successfully incorporated into proteins *in vivo* (Vargas-Rodriguez et al., 2018). MjTyrRS mutants are active toward >50 ncAAs containing either β- or γ-aromatic side chains with functional groups while PylRS mutants are responsible for >100 ncAAs including lysine derivatives containing aliphatic side chains and functional groups, as well as phenylalanine derivatives with aromatic side chains. Noticeably, the recognition profiles of these enzymes overlap on certain phenylalanine analogs. The challenges, methods and future perspectives for directed evolution of o-aaRSs were reviewed elsewhere (Crnkovic et al., 2019) including a powerful evolution strategy using phage-assisted continuous evolution (PACE) (Bryson et al., 2017).

Alternative OTS has been developed for direct encoding of phosphoserine (Sep), which was enlightened by the discovery of an RNA dependent cysteine biosynthesis pathway in archaea (Sauerwald et al., 2005). It takes advantage of a dedicated tRNA^Sep^ (derived from MjtRNA^Cys^) and an optimized *o*-phosphoseryl-tRNA synthetase (SepRS) (Park et al., 2011) to generate the Sep-tRNA^Sep^. The Sep-tRNA^Sep^ can be delivered to amber codon using an engineered EF-Tu albeit with low efficiency. Directed evolution of the SepRS and/or tRNA^Sep^ led to new variants with enhanced Sep incorporation efficiency and was used to produce homogeneously modified proteins in amounts sufficient for biological function analysis (Lee et al., 2013; Rogerson et al., 2015). This orthogonal pair was also evolved for genetic encoding of phosphothreonine (Zhang M. S. et al., 2017).

The engineered tRNA^UTU^/(SerRS, SelA) pair enables site-specific incorporation of selenocysteine (Sec) via amber suppression. This approach bypasses the need for the Sec-dedicated elongation factor SelB and the conserved Sec-insertion sequence element (SECIS) on mRNA, which are required in natural translation of *opal* codon to Sec. The chimera tRNA^UTU^, based on tRNA^Ser^ and tRNA^Sec^, was subjected to a two-step conversion: firstly is aminoacylated by SerRS and then converted to Sec-tRNA^UTU^ by selenocysteine synthase (SelA) (Aldag et al., 2013). The Sec was then delivered to *amber* codon using EF-Tu, instead of SelB. Further efforts generated more effective tRNAs, such as tRNA^UTUX^ (Miller et al., 2015) and tRNA^UTUT6^ (A59C mutation) (Fan et al., 2018), that afford production of proteins with high Sec/Ser ratios (>80%). An alternative effort focused on a newly discovered allo-tRNA family which has unusual acceptor branches acting as efficient serine acceptors. Engineering of the allo-tRNA and SelA from *Aeromonas salmonicida*, as well as the *E. coli* selenium metabolism improved the cellular expression yield and purity (>80%) of recombinant human glutathione peroxidase (Mukai et al., 2018).

##### 3.2.2.2. aaRS and tRNA engineering

The aaRS based aminoacylation approach poses many restrictions on tRNA engineering, as many elements of the latter are involved in the interaction with the former (Figure 4C). Most of the reported o-tRNA engineering focuses on variations of EF-Tu interacting nucleotides. For instance, the directed evolution and/or rational design of MjtRNA^Tyr^ and tRNA^Pyl^ in the T- and acceptor-stem yield optimized tRNAs with better ncAA incorporation efficiency (Guo et al., 2009; Fan et al., 2015).

MjTyrRS is lacking most of the non-conserved domain that binds to the anticodon loop of its cognate tRNA^Tyr^ (Steer and Schimmel, 1999; Kobayashi et al., 2003). As a result it has some promiscuity toward tRNA's anticodon allowing it to be used not only for amber suppression, but also reassignment of *opal, ochre*, sense codons (AGA/U) (Wang and Tsao, 2016; Vargas-Rodriguez et al., 2018) as well as unnatural codons (A*NaM*C) (Fischer et al., 2020). Surprisingly, an engineered initiator tRNA^fMet^ harboring one mutation in the acceptor stem (A72G) and two mutations in the anticodon nucleotides (A35U, U36A) is a substrate of the MjTyrRS for ncAA acylation (Tharp et al., 2019). Despite its low efficiency, this is the first example of o-tRNA/aaRS being used for initiation reassignment. The ncAA incorporation efficiency on AUG start codon is enhanced by deletion of tRNA^fMet^ gene in the *E. coli* genome (Tharp et al., 2019).

The tRNA^Pyl^/PylRS is orthogonal to both *pro-* and *eukaryotic* systems including living organisms as well as CFPSs (Nozawa et al., 2009). The anticodon of tRNA^Pyl^ is not involved in PylRS recognition making this system suitable for genetic code expansion technique, not only for nonsense codons but also for sense, quadruplet codons (Cui et al., 2018; Vargas-Rodriguez et al., 2018; Oller-Salvia and Chin, 2019) as well as unnatural triplets (Fischer et al., 2020). The commonly used PylRS variants originate from *Methanosarcina barkeri* (Mb) and *Methanosarcina mazei* (Mm), comprising N-terminal tRNA binding domains and C-terminal catalytic domains. These enzymes have higher activity than their bacterial counterparts from *Desulfitobacterium hafniense* (Dh) whereby DhPylSn and DhPylSc are expressed separately and gain functionality after assembly *in vivo*. Recently, genome data mining identified a new class of PylRS lacking the N-terminal domain, such as the one from *Methanomethylophilus alvus* (Ma), that is both efficient and orthogonal in *E. coli* (Willis and Chin, 2018). The anticodon stem loop of MatRNA^Pyl^ features a nucleotide bulge. By optimizing the variable loop, an efficient mutant, MatRNA^Pyl(6)^, was obtained that is only accepted by MaPylRS but not MmPylRS resulting in an orthogonal tRNA/aaRS pair (Willis and Chin, 2018).

##### 3.2.2.3. Diversity of genetically encoded ncAAs meditated by o-aaRSs

**Figure 7:**
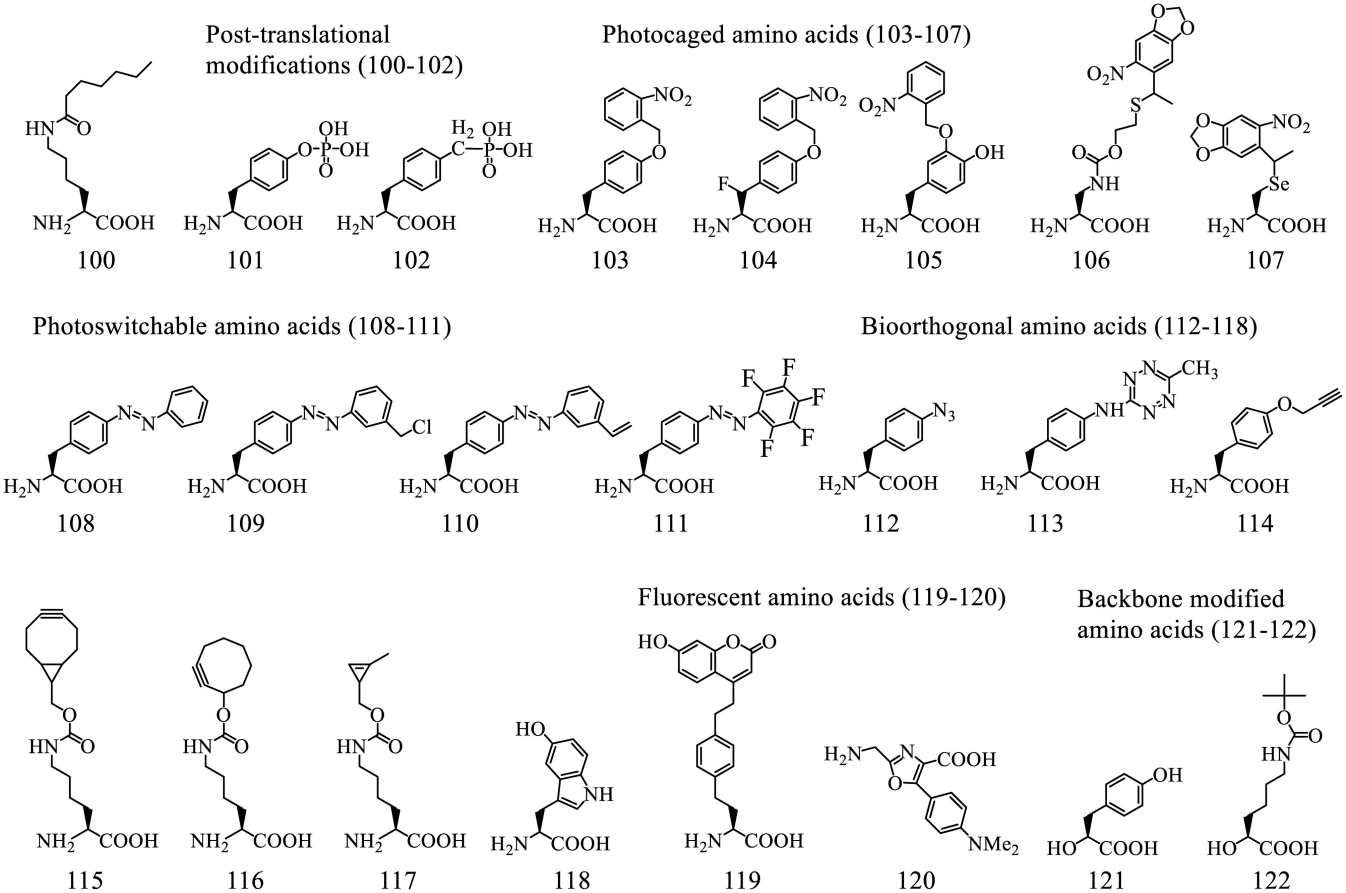
A representative list of ncAAs incorporated into proteins using o-tRNA/aaRS pairs.

The amino acid binding pockets of aaRSs have been subjected to directed evolution to accommodate a range of ncAAs. These ncAAs contain useful functional groups such as post-translational modifications, photoreactive handles, bioorthogonal reactive groups as well as fluorescent groups. A representative list and some recent additions to these ncAAs are shown in Figure 7. Reports of aaRS-mediated incorporation of ncAAs including the ones used as probes for NMR, IR, and crystallographic analysis, used for enhancing the enzymatic activity, enantioselectivity, ancillary function, creating novel metal-binding sites, or making catalytic residues, are reviewed elsewhere (Dumas et al., 2015; Xiao and Schultz, 2016; Yu et al., 2018; Drienovská and Roelfes, 2020).

*3.2.2.3.1. Post-translational modifications (PTMs)*. Post-translational modifications (PTMs), such as Lysine acetylation (Neumann et al., 2009; Lammers et al., 2010), methylation (Nguyen et al., 2009), ubiquitination (Virdee et al., 2010, 2011), can be incorporated into protein sequences either directly or indirectly to facilitate their characterization in various biological process. PTMs could alter the properties of protein/peptide-based therapeutics. A recent example shows the incorporation of ε-N-heptanoyl-l-lysine (HepoK, **100**) that enhances GLP1 peptide binding to human albumin and confers a more potent and long-lasting ability to decrease blood glucose levels (Fu et al., 2019).

**Figure 8:**
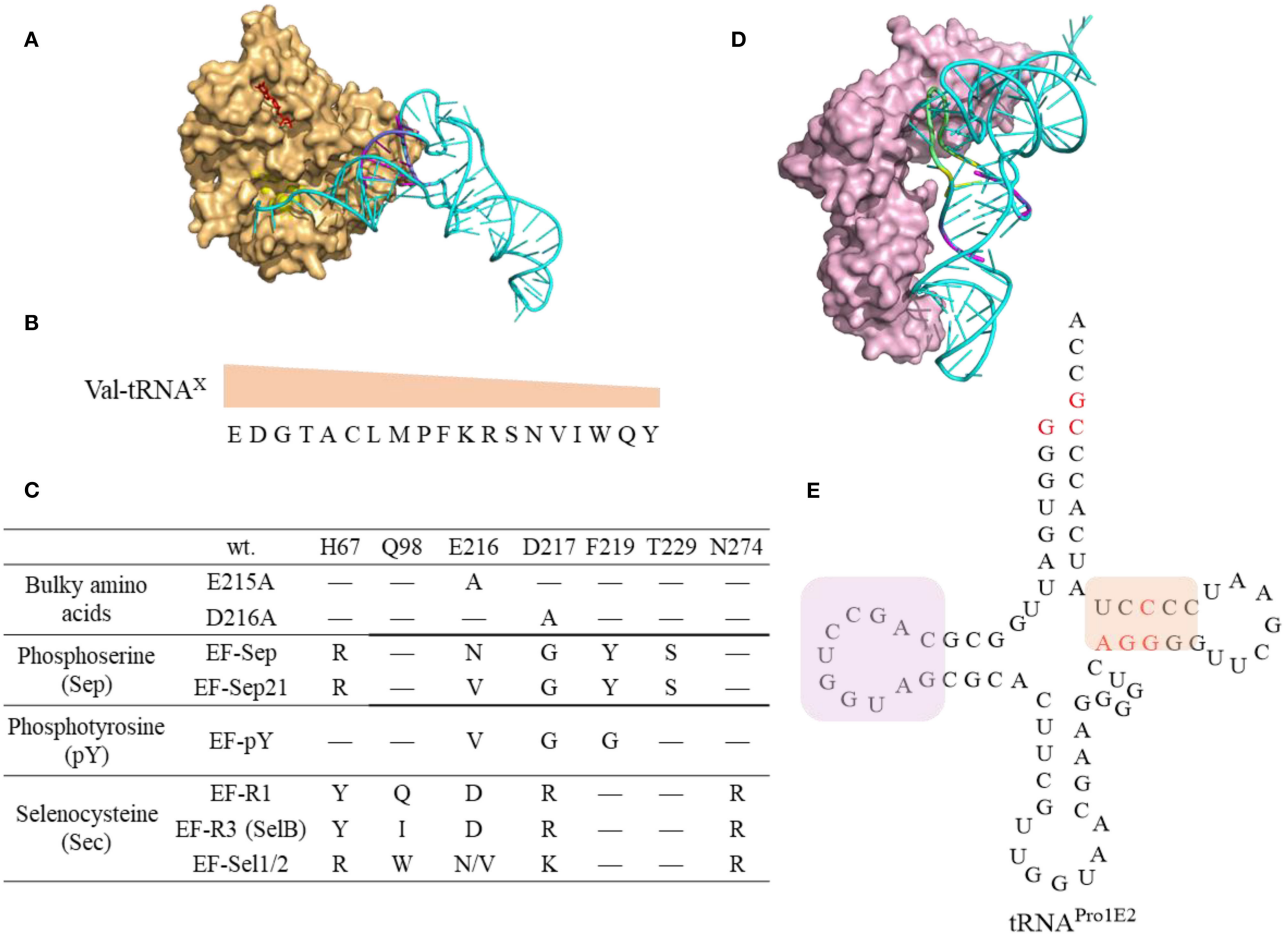
Interaction of tRNAs and elongation factors. **(A)** A representative structure of tRNA bound to an elongation factor Tu (PDB: 5AFI). The EF-Tu is shown as a molecular surface (orange) with the amino acid binding pocket highlighted in yellow and the GDP shown in red. The tRNA (Ec tRNA^Phe^) is shown in cyan with the T-stem colored in magenta which is the main contributor for EF-Tu binding affinity. **(B)** The relative EF-Tu binding affinity toward 19 tRNA isoacceptors precharged with Val. The single letter of amino acid represents the corresponding tRNAs. For example, tRNA^Glu^ charged with Val has the highest binding affinity toward EF-Tu (Asahara and Uhlenbeck, 2002). **(C)** The EF-Tu mutants that improve incorporation efficiency of certain ncAAs. EF-R3 with transplanted residues from SelB of the amino acid binding pocket shows enhanced selenoprotein production activity. **(D)** A representative structure of tRNA interacting with the elongation factor P (PDB: 6ENJ). The D-stem (magenta) and D-loop (yellow) in Ec tRNA^Pro^ (Cyan) are critical structural elements for recruiting EF-P (light pink) to promote peptide bond formation for challenging amino acids. **(E)** The cloverleaf structure of the engineered tRNA^Pro1E2^. The mutations introduced in Ec tRNA^Pro1^ are highlighted in red. This tRNA maintains the original D-stem loop structure for EF-P interaction (framed in pink box) while with an optimized T-arm with enhanced EF-Tu binding affinity (framed in orange box).

A common PTM phosphotyrosine (pTyr, **101**) is a difficult target for direct genetic decoding due to the negative charge of its side chain that causes poor interaction with EF-Tu. Its incorporation was achieved using engineered EF-Tu (Figure 8C) in a phosphatase knockout strain (Fan et al., 2016). Although the yield of pTyr-containing sfGFP is only about 5% that of wild type (wt) sfGFP, 20 mg/L is sufficient for biological studies of tyrosine phosphorylation. Alternative methods circumvent this issue by genetically encoding a non-hydrolyzable analog of pTyr (**102**) (Luo et al., 2017). Although feasible, it sometimes could not fully represent the pTyr due to the minor structural difference.

*3.2.2.3.2. Photo-responsive ncAAs*. Photocaged ncAAs can be used to regulate protein functions by exposure to light, which makes them useful cell-biological tools. For instance *o*-nitrobenzyl-Tyr (**103**) was used to construct light-activatable nanobodies (Jedlitzke et al., 2020) and antibody fragment (Bridge et al., 2019), *o*-nitrobenzyl-4-hydroxyphenylalanine (**105**) was used for generating a photo-controlled variant of wet adhesive protein (Hauf et al., 2017), a caged diaminopropionic acid (**106**) was used for catalytic mechanism characterization of valinomycin synthetase (Huguenin-Dezot et al., 2019), a photocaged Sec, 4,5-dimethoxyl-2-nitrobenzyl-Sec (**107**), was used for production of selenoproteins as well as for site specific alkylation (Welegedara et al., 2018).

Photocaged crosslinkers such as *o*-2-nitrobenzyl-β-fluorotyrosine (FnbY, **104**) were used to cross-link proteins to identify their interacting partners *in vivo* (Liu et al., 2019). Photoswitchable biomolecules, such as phenylalanine-4'-azobenzene (**108**), harboring photoisomerizable functionalities that could induce reversible changes in protein conformation and functionality, offer minimal invasive approach for precise spatiotemporal control. Photoswitchable click amino acids (PSCaa) have an additional click functional group such as benzyl chloride (**109**, trans), alkene (**110**, trans), keto, and pentafluoro PSCaa (**111**, trans), were used to generate a covalent protein bridge by reacting with a nearby cysteine residue through proximity-enabled bioreactivity (Hoppmann et al., 2014, 2015; Klippenstein et al., 2017). The resultant azobenzene bridge isomerized in response to light, thereby changing the conformation of the protein and its activity.

*3.2.2.3.3. Bioorthogonal handles*. Bioorthogonal handles are useful for installation of desired chemical groups, such as small affinity tags, fluorophores, or linkers for protein conjugation (Lee K. J. et al., 2019). The ncAAs with groups such as ketones, azides (**112**), tetrazine (**113**), terminal alkynes (**114**), strained alkynes and alkenes (**115-117**), remain inert in the endogenous biological environment and react with complementary probes. The most popular reaction schemes are copper-catalyzed azide alkyne cycloaddition (CuAAC), photoclick cycloaddition, strain-promoted azide alkyne cycloaddition (SPAAC), and inverse electron-demand Diels-Alder cycloadditions (IEDDA) (Lee K. J. et al., 2019). The IEDDA reactions between electron-poor tetrazines and strained alkyne/alkene moieties, such as bicyclo[6.1.0]nonyne (**115**) (Lang et al., 2012a), trans-cyclooctene (**116**) (Nikic et al., 2014), cyclopropene (**117**) (Elliott et al., 2014), and norbornene (Lang et al., 2012b), are rapid under physiological conditions. A recently developed chemoselective rapid azo-coupling reaction (CRACR) between an aromatic diazonium ion and 5-hydroxyl-Trp (**118**) (Addy et al., 2017) is orthogonal to both SPAAC and IEDDA chemistries. These chemistries were used for installing multiple ncAAs into polypeptides site-specific fashion (Italia et al., 2019).

*3.2.2.3.4. Fluorescent amino acids*. Direct incorporation of fluorescent ncAAs minimizes the functional interference with target proteins, compared to other labelling methods. However, these ncAAs, such as coumarin-derived amino acid (**119)**, suffer from low quantum yield (Wang et al., 2006a; Lee K. J. et al., 2019). A recent report demonstrated genetic encoding in cells with a bright fluorescent oxazole amino acid (**120**) that does not contain either an α-amine moiety or any asymmetric center. This was achieved by using a modified ribosome albeit with low efficiency (Chen et al., 2019).

*3.2.2.3.5. Backbone modified ncAAs*. Unlike the Flexizyme-mediated approach, only a very limited number of backbone-modified amino acids have been installed via o-tRNA/aaRS pairs. These include MjTyrRS for α-hydroxy-L-phenyllactic acid (**121)** (Guo et al., 2008), PylRS for α-hydroxyl acid analog of Boc-lysine (Boc-LysOH, **122**) (Kobayashi et al., 2009) as well as a fluorescent oxazole amino acid (**120**) (Chen et al., 2019). All these ncAAs were incorporated into proteins with very low efficiency (<10%), which might be caused by poor aminoacylation, inefficient EF-Tu interactions, and slow ester/amide bond formation in the ribosomal decoding center.

##### 3.2.2.4. Simultaneous incorporation of multiple ncAAs

Creating mutual orthogonal aminoacyl-tRNA synthetase (aaRS)/tRNA pairs is necessary for faithful installation of multiple ncAAs. The tRNA^Pyl^/PylRS pairs are orthogonal to the MjtRNA^Tyr^/TyrRS and have been used for orthogonal dual incorporation of structurally distinct substrates. For instance, a cyclopropene derivative of Lys (CypK, **117**) and *p*-propargyloxy-phenylalanine (PrpF, **114**) are genetically encoded by MbPylRS-PylT_UACU_ and MjPrpFRS-tRNA^Tyr^CUA, respectively, and were used in phage display for selecting polypeptides with expanded chemistries (Oller-Salvia and Chin, 2019). The Sep OTS is orthogonal to Pyl OTS and their combination was used for simultaneous incorporation of phosphoserine (tRNA^Sep^/SepRS/EF^Sep^) and acetyllysine (tRNA^Pyl^/AcKRS) (Venkat et al., 2018).

Evolution of substrate selectivity of two polyspecific MjTyrRSs, pCNFRS that recognizes >18 ncAAs and AcKRS, generated variants that are highly selective for *p*-azidophenylalanine (**112**) and *m*-iodo-L-phenylalanine, respectively (Kwok et al., 2019). However, using two MjTyrRS derived mutants for simultaneous incorporation of two ncAAs is still problematic due to the lack of orthogonality of the tRNAs. In contrast, tRNA^Pyl^/PylRS pairs of different origins were evolved to be orthogonal to each other and were used for dual site-specific ncAAs incorporation via amber and frameshift suppression using MaPylRS(CbzKRS)/tRNA^Pyl(6)^ and MmPylRS(CypKRS)/tRNA^Pyl^ combination (Willis and Chin, 2018). The active sites of these two PylRS enzymes are specific for two ncAAs, N^ε^-benzyloxycarbonyl-lysine (CbzK) and CypK (**117**) (Willis and Chin, 2018).

Simultaneous incorporation of three ncAAs (**112, 117, 118)** was achieved by combining MjtRNA^Tyr^/TyrRS, MbtRNA^Pyl^/PylRS and EctRNA^Trp^/TrpRS pairs in an *E. coli* ATMW1 strain whereby the endogenous EctRNA^Trp^/TrpRS pair was functionally replaced by its yeast counterpart (Italia et al., 2019). The resulting sfGFP protein contains the most types of genetically encoded ncAAs so far. Although impressive, this strategy relies on reassignment of all three stop codons and one of them is read either as an ncAA or as termination signal. This complicates the procedure and requires aforementioned special design for protein expression.

### 3.3. Engineering Other Elements of Translational Machinery for Enhanced ncAA Incorporation

Not all the proteinogenic amino acids are incorporated into proteins using identical mechanisms. Nature has evolved dedicated elongation factors EF-P and SelB for efficient delivery of consecutive Pro (Doerfel et al., 2013) and selenocysteine (Aldag et al., 2013), respectively. Other modified amino acids, such as phosphoserine, phosphotyrosine as well as many other modifications, are post-translationally introduced into protein sequences. Modification of translational machinery is expected to provide solutions to limitations in incorporating structurally complex ncAAs.

#### 3.3.1. Elongation Factors

The amino acids and tRNA sequences are fine-tuned to ensure appropriate EF-Tu binding affinity and kinetics that sufficient to form ternary complex and yet being able to release EF-Tu for codon decoding (Schrader et al., 2011) (Figure 8A). The thermodynamic contributions of the esterified amino acid and the tRNA to the overall binding affinity are independent and compensate each other (LaRiviere et al., 2001). The binding affinity of tRNA^Glu^ and tRNA^Asp^ to the EF-Tu are among the highest indicating the relatively little contribution from the negatively charged Glu and Asp (Asahara and Uhlenbeck, 2002) (Figure 8B). It is possible to fine tune not only the sequence of the orthogonal tRNA, but also the EF-Tu to achieve the best incorporation efficiency of challenging ncAAs.

EF-Tu has been engineered to display enhanced binding affinity toward challenging ncAAs (Figure 8C). For instance, incorporation of bulky ncAAs, 1-pyrenylalanine, DL-2-anthraquinonylalanine, L-2-pyrenylalanine, which are poorly incorporated into streptavidin via the wt EF-Tu, was achieved by introducing mutations in EF-Tu (E215A or D216A) to expand its amino acid binding pocket leading to incorporation efficiency at 8–25% in PURE system (Doi et al., 2007). An engineered EF-Tu (EF-Sep) containing five mutations in the amino acid binding pocket is capable of mediating incorporation of negatively charged phosphoserine into *amber* codon, albeit with low efficiency (1–25 μg of MEK1 proteins with single or double phosphoserine were produced per liter of *E. coli* culture) (Park et al., 2011). Further optimized SepRS and EF-Sep (EFSep21) afforded production of significant amounts (3 mg per L culture) of recombinant full length phosphohistone H3 (Lee et al., 2013). However, subsequent work on systematic evolution of the anticodon stem loop of tRNA^Sep^, and the anticodon recognition region of SepRS, identified new pairs of tRNA^Sep^/SepRS that have enhanced Sep incorporation efficiency without optimization of EF-Sep interaction (Rogerson et al., 2015). Although many engineered OTSs for Sep incorporation are generated and investigated in the in *vivo* system, these elements could be transferred to the cell-free system. For instance, the cell free extract derived from *E. coli* strains expressing the 1st generation of EF-Sep, SepRS, and tRNA^Sep^ enables production of up to a milligram of phosphorylated human MEK1 kinase (Oza et al., 2015). The produced protein contained some non-phosphorylated species. Using more efficient OTSs might be beneficial for increasing protein purity.

EF-Tu could be engineered to support increased selenocysteine (Sec) protein production either by its directed evolution (EF-Sel) or by rational transplanting of the residues from elongation factor SelB to EF-Tu, in order to generate variants with positively charged amino acid binding pockets (EF-R1, EF-R3) (Haruna et al., 2014). The EF-Sel mutants selected through directed evolution is beneficial for the modified protein yields but not as good as EF-R1/R3 variants, which may represent a compromise between selenoprotein toxicity and yield. Cellular fitness puts restrictions on the range of engineering of the orthogonal translation systems. The efficiency of these *in vivo* engineered EF-Tus is yet to be characterized for *in vitro* protein synthesis.

EF-P could stimulate the formation of the first peptide bond and accelerate synthesis of proteins containing consecutive prolines by preventing ribosomal stalling and promoting peptide bond formation (Blaha et al., 2009; Doerfel et al., 2013). The discovery of recognition elements in tRNA^Pro^ for EF-P recruitment (Katoh et al., 2016) inspired the design of tRNA^ProE2^ (Figure 8E) as aforementioned. EF-P recognizes both the tRNA^Pro^ (Figure 8D) as well as the peptidyl-Pro residue in the P site to accelerate Pro-aa bond formation, where aa is a poor A-site substrate such as Pro, Gly, or other secondary amino acids. Experimental data shows EF-P is not only effective in alleviating the ribosome stalling on the consecutive Pro, but also on D-amino acids (Katoh et al., 2017a), β-amino acids (Katoh and Suga, 2018) and other structurally challenging ncAAs (Katoh et al., 2017b), at optimal concentration of 5–10 μM. Higher concentrations are detrimental possibly due to the longer residence of EF-P in the vicinity of the ribosomal E site thus inhibiting the translocation of deacylated tRNAs from ribosomal P site to E site.

#### 3.3.2. Ribosome Engineering

**Figure 9:**
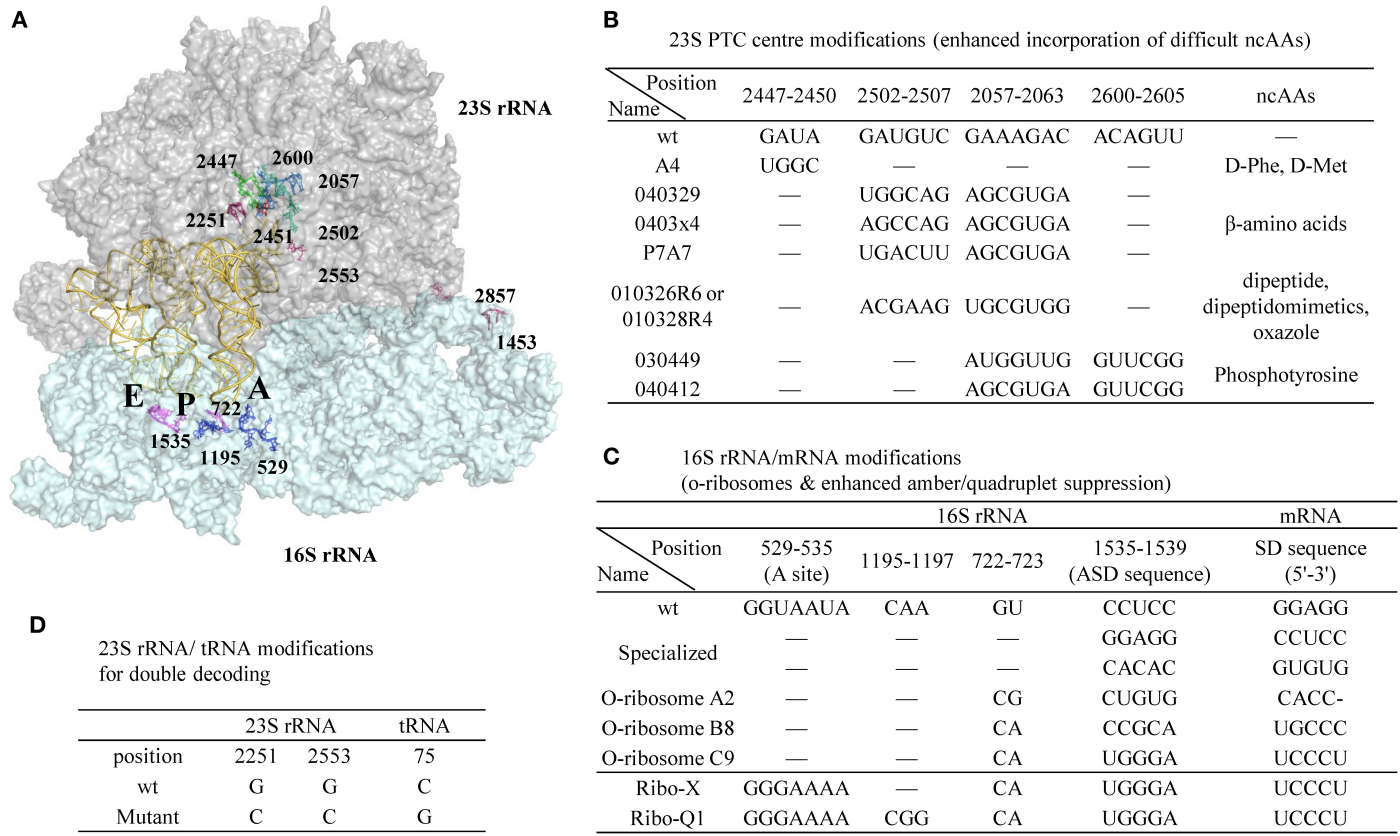
A representative ribosomal RNA structure in complex with A-, P-, and E-site tRNAs and the positions of ribosomal modifications. **(A)** 23S and 16S rRNA are shown as surface colored by gray and cyan, respectively. The tRNAs are shown as cartoon in yellow. **(B)** Modifications in the peptidyl transferase center (PTC) of 30S rRNA enhanced incorporation efficiency of certain challenging ncAAs. **(C)** Modifications in 16S rRNA/mRNA pair to generate orthogonal ribosomes with enhanced amber or quadruplet suppression efficiency with o-mRNA. **(D)** Modifications in 23S rRNA/tRNA pair that creates the second set of genetic code of the same mRNA template in parallel to the wt 23S rRNA/tRNA.

Ribosome engineering continues pushing the limits of this biocatalyst. Recent efforts have focused in several areas: enhancing ribosome ability to polymerize non-native monomers, generating orthogonal ribosomes that function in parallel to the wt systems and hence could be subjected to directed evolution, and creating double genetic codes of the same mRNA templates to increase the information encoding density (Figure 9).

Point mutations in the peptidyl transferase center (PTC) of 23S rRNA enhanced D-amino acid incorporation efficiency (Dedkova et al., 2003, 2006). Compared to wt ribosome, the S30 extract prepared from the cells containing both the wt and a mutant (A4, Figure 9B) has increased yield of proteins harboring D-Phe or D-Met from 3–5% to 12–23%. Noticeably, alteration of a single nucleotide in the key position of the ribosomal PTC can be lethal to the cells, while multiple mutations can rescue activity by maintaining general PTC architecture (Thompson et al., 2001). Due to the opposite chirality of the C^α^-atoms, the α-amino group of the D-amino acid is positioned further away from the peptidyl-tRNA carbonyl group carbon in the wt ribosome P-site, thus resulting in non-optimal nucleophilic attack and blocking proton shuttling during the peptide bond formation (Melnikov et al., 2019). The A4 mutant has four-nucleotide changes at position 2447-2450 and is expected to lead to a larger A-site cleft that is beneficial for D-amino acid incorporation.

The reorganization of ribosomal PTC architecture through mutagenesis of two regions in 23S rRNA, instead of simple mutations in one region, is effective in decoding other structurally challenging ncAAs (Figure 9B). Two of the modified ribosomes (040329 and 0403x4) were able to suppress UAG codon in *E. coli* producing β-Ala modified proteins (Dedkova et al., 2012) with suppression efficiency of ~10–15% compared to 4% of wt ribosome. The same research group succeeded in incorporation of various β-amino acids, but all with <20% efficiency (Maini et al., 2013). Directed evolution also identified ribosomes that are capable of accepting dipeptides, dipeptide analogs, oxazole fluorescent amino acid (010326R6/010328R4) (Maini et al., 2015; Chen et al., 2019) as well as phosphorylated tyrosine (030449 and 040412 mutants) (Chen et al., 2017), as substrates.

Another direction in ribosome engineering is the generation of orthogonal ribosomes that operate as independent translation systems translating target proteins while the wt ribosomes continue to synthesize the genome-encoded proteins to ensure cell viability (Figure 9C). This is achieved by co-modifying the anti-Shine Dalgarno in the 16S rRNA as well as the Shine Dalgarno sequence in the engineered mRNAs to enable gene-specific translation (Hui and de Boer, 1987; Rackham and Chin, 2005a). Combination of the three o-ribosome (A2, B8, C9)/o-mRNA pairs to control downstream translation of variable protein fragments enables creation of combinatorial logic gates in the living cells, and opens the door for generating cellular computers controlled by the biomolecules (Rackham and Chin, 2005b).

Further optimization of the orthogonal ribosome A-site located at the 530 loop of the 16S rRNA was performed to reduce its interaction with RF1 thereby enhancing suppression efficiency (Wang et al., 2007). The evolved o-ribosome termed Ribo-X, with two-nucleotide mutation in the loop region, demonstrated 60% and 20% suppression efficiency toward an o-mRNA that harbored one and two amber codons, respectively, to *p*-benzoyl-L-phenylalanine. Much larger libraries covering 127 residues within 12Å of the tRNA bound in 16S rRNA decoding center were generated, and the evolved orthogonal ribosome Ribo-Q1 could efficiently decode both quadruplet (AGGA) and amber codon (Neumann et al., 2010). This modified ribosome has been recently used to incorporate CypK (**117**) and PrpF (**114**) at two sites of ScFv followed by subsequent conjugation with two distinct fluorophores (Oller-Salvia and Chin, 2019).

In order to restrict the exchange of the large subunits between pools of the native and orthogonal small subunits, the later versions of orthogonal ribosomes (Ribo-T) were generated where the small and large subunits were tethered together via helix 44 of the 16S rRNA and helix 101 of the 23S rRNA using either polyadenine linkers or RNA hinges from self-splicing introns [16S(1-1453)-linker-23S(2858-2902-1-2857)-linker-16S(1454-1542)] (Fried et al., 2015; Orelle et al., 2015; Schmied et al., 2018) (Figure 9A, reconnecting position at 1453 in 16S rRNA and 2857 in 23S rRNA). These new ribosomes enable selection of mutants 30S subunit capable of re-programming cellular logic (Rackham and Chin, 2005b) and enabling new decoding properties (Wang et al., 2007; Neumann et al., 2010). However, the altered arrangement of rRNA sequences with a permutated 23S rRNA inserted into 16S rRNA reduced their ability to rapidly assemble into a macromolecular machine. The 2nd generation of Ribo-T was evolved by selecting new RNA tethers from a more diversified library that connect the 16S and 23S rRNA (Carlson et al., 2019). Ribosome profiling of Ribo-T cells shows no defects in translation elongation but reveals somewhat higher occupancy by Ribo-T of the start codons and to a lesser extent stop codons, suggesting that subunit tethering mildly affects the initiation and termination stages of translation.

Two concomitantly functioning orthogonal translation machineries can be achieved not only by manipulating the aforementioned mRNA/16S rRNA pair, but also by engineering the tRNA/30S rRNA recognition patterns (Terasaka et al., 2014). Two highly conserved residues G2251G2252 at P-site of the 23S rRNA interact with C75C74 (tRNA) via Watson-Crick base paring while another nucleotide G2553 in A-site base pairs C75 of the incoming tRNA. Engineering these conserved nucleotides in the peptidyl transferase center in 23S rRNA as well as the ones in tRNA 3'-CCA-end demonstrated that the compensatory restoration of these base pairs recovers the translational activity. Two mutant ribosome-tRNA pairs were found to be orthogonal to the wt pair. One of these pairs (23S rRNA-G2251CG2553C/tRNA-C75G) demonstrated good activity and functioned together with the wt pair in the translation reaction (Figure 9D). They acted orthogonally and translated the same mRNA sequence into two distinct peptide sequences (Terasaka et al., 2014).

## 4. Genome Wide Codon Engineering

A genomically recoded *E. coli* strain (C321.ΔA), where all the 321 UAG *amber* stop codons were replaced by synonymous UAA codons, permitted the deletion of RF1 and more efficient reassignment of UAG codons (Lajoie et al., 2013). This strain has served as a synthesis platform for modified proteins such as phosphoproteins (Pirman et al., 2015), as well as a platform for enzyme evolution (Amiram et al., 2015). In the latter case, chromosomally integrated aaRS variants based on this strain have been evolved to enable multi-site ncAAs incorporation. Despite the lack of competition from the RF1 and superior ability for amber suppression, this strain grows slower and sometimes produces less modified proteins than BL21(DE3) (George et al., 2016). An unexpected codon skipping phenomenon was observed in this strain when producing Sep-ubiquitin proteins that missing one amino acid residue, the ncAA, with the +3 frameshifting (George et al., 2016). The mechanism behind this phenomenon is unexplored.

The C321.ΔA strain can serve as a chassis strain for crude CFPS. The cell extract from this strain integrated with an improved Sep-OTS components enable production of phosphoserine-protein at mg per ml yield (Oza et al., 2015). However, the standard cell extract from this strain supports the production of wt proteins with about 3 times lower efficiency than that of the standard BL21(DE3) strain (Martin et al., 2018). Inactivation of negative effectors in the host strain using multiplexed automated genome engineering (MAGE) generated a new strain, C321.ΔA.759 (*endA-, gor-, rne-, mazF-*). The derived cell extract from this strain has improved productivity for both wt and modified proteins (Martin et al., 2018). With an increasing number of UAG codon present in the sfGFP template, an exponential decrease of protein yield was observed for the cell extract derived from BL21Star (DE3), leading to no detectable active protein for sfGFP-5UAG. The yield from release factor 1 (RF1) deficient MCJ.559 strain in which only a small set of essential genes were recoded (Hong et al., 2014b), is roughly 2 times less compared to the one from C321.ΔA.759. Incorporation of 8 and 9 consecutive ncAAs is also achieved with the full-length protein ratio at 75 and 60%, respectively. Keeping a proper surface area to volume ratio, the batch translation reaction could be scaled up to 17-fold from 15 to 255 μl without loss of productivity (Martin et al., 2018).

Genomic engineering of *E. coli* strains free of sense codons was also attempted. The first ambitious project to build a 57-codon *E. coli* was reported in 2016, which aimed to replace 7 codons (R-AGG, R-AGA, S-AGC, S-AGT, S-TCG, S-TCA) with their synonymous codons (Ostrov et al., 2016). However, it turned out that replacement of 13 out of 123 AGR codons to the synonymous codon CUG was detrimental to the cells, requiring diversification to viable codon alternatives (Napolitano et al., 2016). It is not surprising that drastic change in the genome structure might cause cell fitness issues.

Impressively, recent effort in the Chin lab demonstrated successful deletion of 3 of the 64 codons (amber-TAG, S-TCG, S-TCA) from *E. coli* genome and their replacement with synonymous codons, creating the 61-codon bacteria (Syn61) (Fredens et al., 2019). Due to the absence of sense codons TCG and TCA, deletion of the genes of their cognate tRNAs (*serU* and *SerT*) from the genome is non-lethal. Co-translational incorporation of ncAAs through TCG codon reassignment was achieved using tRNA^Pyl^ CGA/PylRS pair in Syn61, but not in the non-recoded MDS42 strain. This is expected that this pair of OTS leads to wrongly synthesized proteome which is toxic to the standard cells. The Syn61 strain enables site-specific sense codon reassignment without scrambling the proteome and represents a new synthetic biology platform for synthesis of polymers with enhanced and novel activities.

## 5. Summary and Perspectives

We reviewed technical elements for ncAA incorporation ranging from creation of vacant codons, synthesis of ncAA-tRNAs, to translational machinery engineering. These strategies and elements are generally applicable in both types of *E. coli* CFPS, i.e., crude and PURE CFPS. However, due to their distinct compositions, cost and ease to manipulate the translational components, these two *in vitro* translation platforms have advantages and disadvantages for different applications (Table 1).

PURE is the preferred system for peptide synthesis. Impressive work on the genetic reprogramming of the sense codons using this system integrated with Flexizyme tRNA acylation system reveals the extraordinary flexibility of ribosomal synthesis (Figures 5, 6). This affords production of peptides with high diversity and forms an extraordinary screening platform when integrated with mRNA display. However, due to its low productivity and high cost, characterization of the selected non-standard peptide hits relies on chemical synthesis adding an extra layer of complexity. With advances in expression productivity, S30 CFPS might be a promising platform that could combine selection and small-scale production of peptides with ncAAs. This however requires development of approaches for increasing the stability of mRNA and the product peptides.

Substrate promiscuity of the ribosomes is much higher than that of aaRSs. Therefore, the ability to synthesize the ncAA-tRNA largely determines the diversity of novel chemistries that can be incorporated into the polypeptides sequences. Flexizyme represents one solution to this problem and is a very versatile *in vitro* acylation tool that dominates the *in vitro* acylation landscape. The OTSs (o-tRNA/aaRS pairs) are capable of co-translational aminoacylation and have superior capacity to support protein synthesis both *in vitro* and *in vivo* achieving relatively high yields. The Flexizyme can recognize practically any tRNAs with a 3'-DCCA- end, while the aaRS requires many nucleotide identity elements for efficient aminoacylation thereby complicating tRNA engineering. The recently developed T-boxzyme, comprising a tRNA recognition and a catalytic domain, showcased its capacity of co-translational acylation of tRNA^Gly^GCC achieving one-pot *in vitro* synthesis of unnatural peptides (Ishida et al., 2020). Although in its infancy, this T-boxzyme holds promise to transform the tRNA acylation technology and to dramatically expand the structural diversity and increase the productivity of ncAAs-proteins, not only *in vitro* but also in cells.

Reassignment of initiation codon supported by Flexizyme-mediated acylation affords incorporation of more exotic substrate structures such as peptides and foldamers. This is due to the intrinsically large flexibility of the ribosomal initiation P-site. These substrates are too bulky to fit in the ribosomal A-site for elongation. This is consistent with the fact that the dipeptide incorporation via elongation reassignment requires modified ribosomes (Maini et al., 2015). In contrast, initiation reassignment via OTS is far less sophisticated that only one recent example demonstrated its feasibility using an o-aaRS and an engineered initiator tRNA^fMet^ (Tharp et al., 2019). This work demonstrates unexpected versatility of aaRSs. As the initiation accepts a more versatile structures than elongation, it is exciting to see how far the OTSs can reach toward the diversity of the ncAAs.

Poor ribosomal synthesis of proteins with ncAAs might be due to the inefficient binding of ncAA-tRNAs to the EF-Tu and/or compatibility issue in the ribosomal decoding center. Engineering of the translational components is an effective approach to increase the incorporation efficiency of structurally challenging ncAAs. Transfer RNA engineering is beneficial to D-, β-, and many other ncAAs by enhancing EF-Tu binding affinity and/or recruiting of EF-P to facilitate the peptide bond formation. Modified EF-Tu variants have increased capacity for bulky amino acids, negatively charged amino acids including Sep, phosphotyrosine, selenocysteine, but not for D-amino acids. Ribosomes with modified PTCs show beneficial for D-, β-amino acids, dipeptide, fluorescent amino acids, as well as phosphotyrosine. Ribosomes with altered A-site could enhance the *amber* and quadruplet suppression by reducing their interaction to RF1.

Noticeably, the effect of tRNA engineering is specific toward the ncAAs while mutations introduced in EF-Tu and ribosomes cause global effect not only for ncAAs but also for the other standard amino acids in the polypeptide sequences. For instance, the EF-Tu mutants with enhanced D-amino acid incorporation efficiency actually only retain 20–80% activity of wt EF-Tu for canonical amino acids (Doi et al., 2007). Therefore, the benefit brought by the mutated EF-Tu and ribosomes should be balanced when generating proteins containing both canonical amino acids and ncAAs. On the other hand, it seems promising to engineer EF-Tu or ribosome specialized for production of a single polymer type, such as polyesters, peptoids etc. However, current ribosome engineering is generally confined in the *in vivo* system that is governed by cell fitness, ncAA permeability and stability, that set significant limitations. From this perspective *in vitro* translation systems appear as much better platforms for selection of ribosomes capable of synthesizing non-peptide polymers (Hammerling et al., 2019).

Nature has inspired many elegant strategies for enhancing ncAA incorporation. For instance, the direct genetic encoding of phosphotyrosine and phosphothreonine was advanced by the discovery of the RNA dependent cysteine biosynthesis pathway. Consecutive incorporation of D- and β-amino acids were dramatically improved by deciphering of the identity elements in tRNA for recruiting EF-P elongation factor. Discovery of the ΔNPylRS facilitates the evolution of mutually orthogonal tRNA/PylRS pairs. Nature has also evolved strategies to diversify the ribosomal produced polypeptides through post-translational modifications that confer them with a range of added structural and functional features, such as introducing diverse and multiple α-keto-β-amino acids into proteins (Morinaka et al., 2018). Therefore, exploring the diversity of translational components and post-translation modifications from the three kingdoms of life provide sources of further increase in polypeptide chemical diversity.

Using genomically recoded bacteria free of amber codons, RF1 coding genes, as well as several negative effectors, for generating cell free extract enables high yields of proteins containing up to 40 ncAAs with no observable truncated products (Martin et al., 2018). The new genomically recoded organisms free of amber and two Ser codons (Fredens et al., 2019) can serve as a chassis strain for the development of highly efficient CFPS systems capable of site specific incorporation of two or more distinct ncAAs into a single protein or sequence-defined polymers. Utilization of non-natural codons with orthogonal nucleotides beyond the AT(U)GC bases holds the potential to significantly increase the information storage capacity of genes and mRNAs thereby transforming the field of synthetic biology (Zhang Y. et al., 2017; Fischer et al., 2020).

## Data Availability Statement

The original contributions presented in the study are included in the article/Supplementary Material, further inquiries can be directed to the corresponding author/s.

## Author Contributions

All authors listed have made a substantial, direct and intellectual contribution to the work, and approved it for publication.

## Conflict of Interest

The authors declare that the research was conducted in the absence of any commercial or financial relationships that could be construed as a potential conflict of interest.
